# Compartmentalization of bacterial and fungal microbiomes in the gut of adult honeybees

**DOI:** 10.1038/s41522-021-00212-9

**Published:** 2021-05-07

**Authors:** Matteo Callegari, Elena Crotti, Marco Fusi, Ramona Marasco, Elena Gonella, Ivano De Noni, Diego Romano, Sara Borin, George Tsiamis, Ameur Cherif, Alberto Alma, Daniele Daffonchio

**Affiliations:** 1 Biological and Environmental Sciences and Engineering Division (BESE), Red Sea Research Center (RSRC), King Abdullah University of Science and Technology (KAUST), Thuwal, Saudi Arabia; 2 Dipartimento di Scienze per gli Alimenti, la Nutrizione e l’Ambiente (DeFENS), Università degli Studi di Milano, Milan, Italy; 3 Dipartimento di Scienze Agrarie, Forestali e Alimentari (DISAFA), Università degli Studi di Torino, Grugliasco, Italy; 4 Department of Environmental Engineering, University of Patras, Agrinion, Greece; 5 Institut Supérieur de Biotechnologie Sidi Thabet (ISBST), BVBGR-LR11ES31, Biotechpole Sidi Thabet, University Manouba, Ariana, Tunisia; 6 Present Address: School of Applied Sciences, Edinburgh Napier University, Edinburgh, UK

**Keywords:** Microbiome, Microbial ecology

## Abstract

The core gut microbiome of adult honeybee comprises a set of recurring bacterial phylotypes, accompanied by lineage-specific, variable, and less abundant environmental bacterial phylotypes. Several mutual interactions and functional services to the host, including the support provided for growth, hormonal signaling, and behavior, are attributed to the core and lineage-specific taxa. By contrast, the diversity and distribution of the minor environmental phylotypes and fungal members in the gut remain overlooked. In the present study, we hypothesized that the microbial components of forager honeybees (i.e., core bacteria, minor environmental phylotypes, and fungal members) are compartmentalized along the gut portions. The diversity and distribution of such three microbial components were investigated in the context of the physico-chemical conditions of different gut compartments. We observed that changes in the distribution and abundance of microbial components in the gut are consistently compartment-specific for all the three microbial components, indicating that the ecological and physiological interactions among the host and microbiome vary with changing physico-chemical and metabolic conditions of the gut.

## Introduction

Recent studies on the role of bacterial symbionts of the honeybee gut have shed light on their importance for host metabolism, biomass gain, pathogen resistance, and behavioral health^1–4^. The gut bacterial microbiome of adult *Apis mellifera* has extensively been investigated, with a particular emphasis on the taxonomy^3,5,6^, evolution^2^, genomics, and physiology of its bacterial members^1,4,7–9^, as well as on its modification under different environmental conditions, diet, and abiotic and biotic stressors^10–15^. The core gut microbiota of the adult honeybee is composed of five consistent, ubiquitous, and abundant bacterial taxa^16^: the beta proteobacterium *Snodgrassella alvi* (hereafter indicated as *Snodgrassella*), the gamma proteobacterium *Gilliamella apicola* (hereafter indicated as *Gilliamella*), *Lactobacillus* Firm-4, *Lactobacillus* Firm-5, and the actinobacterium *Bifidobacterium*^3,5,17–19^. In addition, the proteobacteria *Frischella perrara* and *Bartonella apis* (hereafter indicated as *Frischella* and *Bartonella*, respectively), together with *Bombella*, *Commensalibacter*, *Apibacter*, and *Lactobacillus* Firm-3, are present, albeit in a lower proportion (~1%–7% of the total bacterial community) in comparison with the former five^5,17,20–22^. These aerobic and facultative anaerobic phylotypes—rarely found outside the bee gut and hive environment^23,24^ and constituting the dominant members (95%–99% of the total bacterial community) of the honeybee gut microbiota—form spatially explicit patterns along the different gut compartments^3,5,25^.

Under natural conditions, bacteria form complex communities, and their evolution, functionality, and ecology are dictated by environmental factors and microbial interactions with host and among microbes^18^. Despite a simple structure and limited number of bacterial species, honeybee gut microbiota exhibit high genomic diversity^18,26^, which may act as a reservoir of metabolic functions that modulate host physiology and nutrition. This metabolic vicariance is encoded by microbial functional redundancy (i.e., each metabolic function can be performed by multiple coexisting and taxonomically distinct organisms) with respect to a multitude of functions, which could induce gut stability and allow bees to adapt to changing environmental conditions (i.e., disturbance)^27,28^. Although such genomic diversity and its localization along the gut are well known for the core microbiome^18^, they are not well characterized for the other microbiome components. For instance, bacteria of environmental origin (e.g., floral nectar and pollen^29^) found in small proportions (1%–5%) of the total bacterial community and having less prevalence (1%–20% of bacterial individuals) are often found in the honeybee gut^5,17,30,31^; however, their compartmentalization along the gut is unknown. Similarly, the compartmentalization of non-pathogenic fungal members occurring in the adult honeybee gut have been rarely investigated. Although several studies have demonstrated by cultivation-dependent and cultivation-independent approaches the presence of yeasts (subphylum *Saccharomycotina*) or other fungal phylotypes (members of *Wickerhamomyces*, *Pleosporales*, and *Agaricales*, among others)^32–38^, only one study analyzed their distribution along the gut^38^.

In the present study, we hypothesized that the honeybee gut compartments (i.e., the crop, midgut, ileum, and rectum) play an important role in mediating the distribution of non-core microbial members (i.e., other potential environmental bacteria and fungi), as they do for core microbial members, in the context of their spatially explicit patterns and different physico-chemical conditions (e.g., O_2_, pH, and redox) of gut. We characterized the microbiota (bacteria and fungi) diversity in the four gut compartments (the crop, midgut, ileum, and rectum) along with changes in gut metabolites, oxygen concentration, pH, and redox potential using a combination of molecular, biochemical, and microsensor approaches. Various analytical methods, including some that were previously used^39^, guided a focused investigation of honeybees collected from their environment (i.e., hives located in flowered prairies) to explore the natural microbial variability of the gut microbiome. This approach will allow us to confirm the presence and compartmentalization of the core bacteria that are consistently associated with the forager honeybee gut and reveal the way in which this spatially explicit pattern is conserved for bacteria and fungi acquired from the environment and not belonging to the core.

## Results and discussion

### Compartmentalization of the bacterial community present in the honeybee gut

**Fig. 1:**
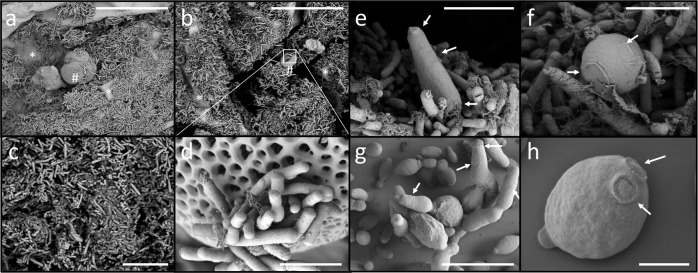
Visualization of microorganisms inhabiting honeybee guts. **a**–**d** Scanning electron microscope (SEM) micrographs showing bacterial cells present in a honeybee gut (i.e., rod-shape bacteria). Grains of pollen covered by bacteria are shown (see also Supplementary Fig. S1). **e**, **f** Fungal cells (i.e., yeast) associated with the gut wall of a honeybee. **g**, **h** Culture of yeast cells isolated from the honeybee gut. Cells belong to *Hanseniaspora uvarum* (strain L18) and *Starmerella bombicola* (strain L28; see also Supplementary Fig. S9 and Supplementary Table S7). Note the different scales on the SEM photographs. Honeybees used for SEM analysis were *A. mellifera jemenitica* from Saudi Arabia (Supplementary Table S6). Scale bars correspond to (**a**, **b**) 30 µm, (**c**) 3 µm, (**d**, **f**) 2 µm, (**e**, **g**) 10 µm, and (**h**) 1 µm.

**Fig. 2:**
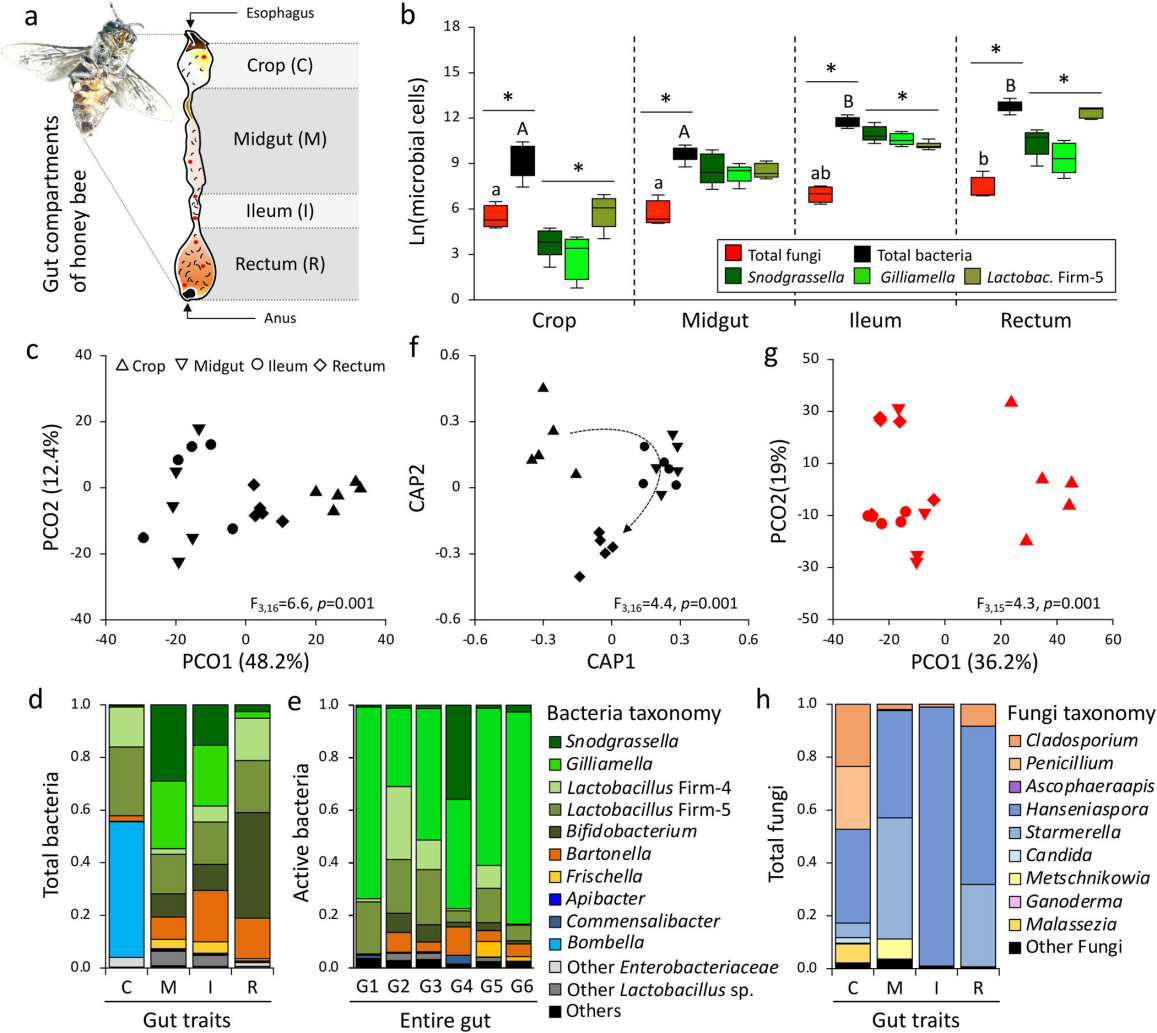
Bacterial and fungal microbiota of the gut compartments in honeybees. **a** Schematic representation of honeybee gut compartments (the crop, midgut, ileum, and rectum). Red circles and black rods indicate the relative distribution of total fungi and bacteria, respectively. **b** The abundance of the bacterial (black bars) and fungal (red bars) members in the honeybee gut compartments are indicated as ln-transformed number of bacterial cells (number of 16S rRNA gene copies measured are normalized for the number of 16S rRNA genes in a bacterial community, *n* = 4.7) and fungal cells (number of measured internal transcribed spacer [ITS] copies are normalized for the number of ITSs in a fungal community, *n* = 75.5). Significant differences among the bacterial and fungal cell abundance along gut tracts are indicated with capital and lower-case letters, respectively (Tukey’s multiple comparison test, *p* < 0.05), whereas the comparison among bacteria and fungi in each trait is indicated by an asterisk (*; *t*-test, *p* < 0.05). Abundance (ln-transformed) of *Snodgrassella*, *Lactobacillus* Firm-5, and *Gilliamella* in the different gut compartments are represented by shades of green and are expressed as the number of bacterial cells (number of 16S rRNA gene copies measured are normalized for the number of 16S rRNA gene in these phylotypes, *n* = 4). Significant differences among the above-mentioned bacterial species for each gut tract are indicated by an asterisk (*; ANOVA, *p* < 0.05). All the results are expressed per organ. Principal coordinates analysis (PCoA) showing the (**c**) bacterial and (**g**) fungal communities per each gut compartment, respectively. **f** Beta-diversity analysis of the other potential environmental bacteria portion. Samples were distributed following a “horseshoe shape” ordination (indicated by the arrow) in the space of the canonical analysis of principal coordinates (CAP). **d**, **e** Taxonomic affiliation of total (DNA) and active (cDNA) bacterial communities inhabiting the honeybee gut, respectively, along with (**h**) one for fungal communities (DNA). Abundance of bacterial taxa obtained from DNA are expressed as the number of reads normalized for the mean number of bacterial 16S rRNA genes and fungal ITSs available for each genus/class, respectively (for details, see “Material and methods”). **b**–**d**, **f**–**h** are referred to the gut compartments’ pools (each pool, *n* = 10) originating from Italian *A. mellifera ligustica* forager bees, whereas **e** is referred to the entire gut of Saudi Arabian *A. mellifera jemenitica* (*n* = 6). Compartmentalization of bacterial and fungal communities associated with Saudi Arabian forager bees is reported in Supplementary Fig. S5.

**Fig. 3:**
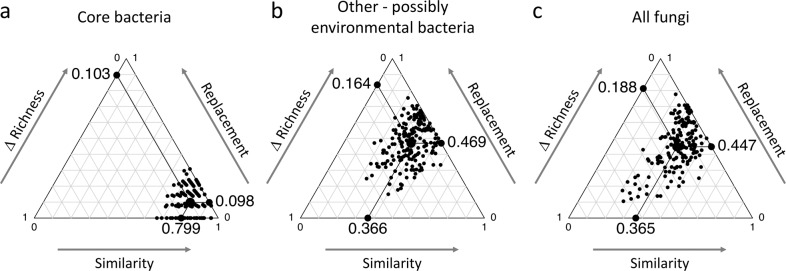
Components of the microbial beta-diversity pattern. Ternary plots are used to quantify the component of the assembly along the gut compartment for (**a**) core bacteria, (**b**) other-possibly environmental bacteria, and (**c**) fungi; note the dataset presented in Fig. 2c, f, g, respectively, were used to perform this analysis. Each point in the triangle plot was determined by a triplet of values from the similarity, replacement, and richness difference components. In each ternary, large central dots from which the lines start represent the centroid of the points; the lines represent the mean values of the three components (i.e., similarity, replacement, and richness difference).

Recent studies have investigated the diversity of the honeybee gut^5,40^ or body^41^ by focusing on the compartmentalization of the main bacterial phylotypes along the intestine^38,42–44^ and overlooking the remaining microbiome components (i.e., fungal and other-possibly environmental bacterial members). In our honeybees, magnified images of the gut indicated the presence of bacterial cells of different morphologies (primarily rod- and coccus-shaped bacteria), colonizing the gut wall and ingested pollen grain surface (Fig. 1a–d and Supplementary Fig. S1). Bacterial quantification (based on a normalized number of 16S rRNA gene copies) showed a significant compartmentalization of their load along the gut (Fig. 2a, b and Supplementary Table S1), with the highest densities observed in the distal compartments (ileum = 1.43 × 10^5^ and rectum = 4.23 × 10^5^ bacterial cells) than in the proximal compartments (crop = 1.83 × 10^4^ and midgut = 1.87 × 10^4^), thereby confirming the data reported in previous studies^21,42^. Furthermore, the gut compartments harbored significantly different bacterial communities (*F*_3,16_ = 6.6, *p* = 0.001; Fig. 2c and Supplementary Table S2) that were characterized by unique bacterial taxa assemblages (Fig. 2d and Supplementary Data 1) with similar alpha diversity (richness and Shannon diversity; *F*_3,16_ = 3.2, *p* = 0.06, and *F*_3,16_ = 0.55, *p* = 0.65, respectively; Supplementary Fig. S2a, b), resulting in a decrease in similarity with the increase in the gut distance (*n* = 190, *p* < 0.0001, *R*^2^ = 0.14; Supplementary Fig. S3a). The bacterial phylotypes belonging to the corbiculate core (i.e., *Snodgrassella*, *Gilliamella*, *Lactobacillus* Firm-4, *Lactobacillus* Firm-5, and *Bifidobacterium*) dominated the gut bacterial community (Fig. 2b, d), as confirmed via amplification of the total-active bacterial community from cDNA (Fig. 2e). The members of the corbiculate core were followed by “*Apis*-specific” *Frischella* and *Bartonella* phylotypes, along with the other corbiculate-associated *Bombella*, *Commensalibacter*, and *Apibacter* (Fig. 2d, e). Collectively, these taxonomic groups—represented by 32 operational taxonomic units (OTUs; Supplementary Fig. S4, Supplementary Data 1, and Supplementary Table S3)—accounted for 92%–98% of the total bacterial community (94%, 93%, 92%, and 98% in the crop, midgut, ileum, and rectum, respectively; Table 1). Consistent with the previous observations^42–44^, a compartment-specific distribution of three of the most abundant core phylotypes was observed (*F*_3,16_ = 10.1, *p* = 0.001); for instance, *Snodgrassella*, *Gilliamella*, and *Frischella* preferentially colonized the midgut and ileum, whereas *Lactobacillus* Firm-4 and Firm-5 mainly colonized the crop and rectum (Fig. 2b, d). Although the abundance, dominance, and ratios of core phylotypes were different^8,17,23^, they were consistently detected along the gut (Fig. 2b, d and Supplementary Table S1). Consequently, the differential richness of the bacterial component was the main determinant of the total beta-diversity pattern of the core gut microbiome of honeybees (Fig. 3a).

**Table 1:** Occurrence of bacterial taxa along the gut compartments of *A. mellifera ligustica* forager bees from Italy.

Bacterial categories	Bacterial taxa	N. OTUs in gut compartment (*n* = 5)				N. OTUs	Abund. (%)
		Crop	Midgut	Ileum	Rectum		
Corbiculate core	*Bifidobacterium*	1	2	2	3	3	15
	*Gilliamella*	4	4	4	4	4	13.2
	Firm-4	3	3	3	3	3	10.6
	Firm-5	9	8	9	9	9	21.1
	*Snodgrassella*	3	3	3	2	4	12.7
*Apis*-specific	*Bartonella*	2	2	2	3	3	10.4
	*Frischella*	1	1	2	1	2	2.2
Other corbiculate associates	*Commensalibacter*	2	2	2	2	2	0.2
	*Apibacter*	1	1	1	1	1	0.2
	*Bombella*	1	1	1	1	1	8.8
Other potential environmental	Other *Enterobacteriaceae*	9	16	15	13	16	3.4
	Other *Lactobacillaceae*	20	11	9	10	21	1.9
	Other Bacteria	80	72	68	51	127	0.8

Operational taxonomic units (OTUs) for each phylotype belong to the corbiculate core, *Apis*-specific, other corbiculate-associated, and other-possibly environmental categories along the four compartments. The total number of OTUs per bacterial taxa and their normalized-relative abundance (abund. %) in the total dataset are reported.

**Fig. 4:**
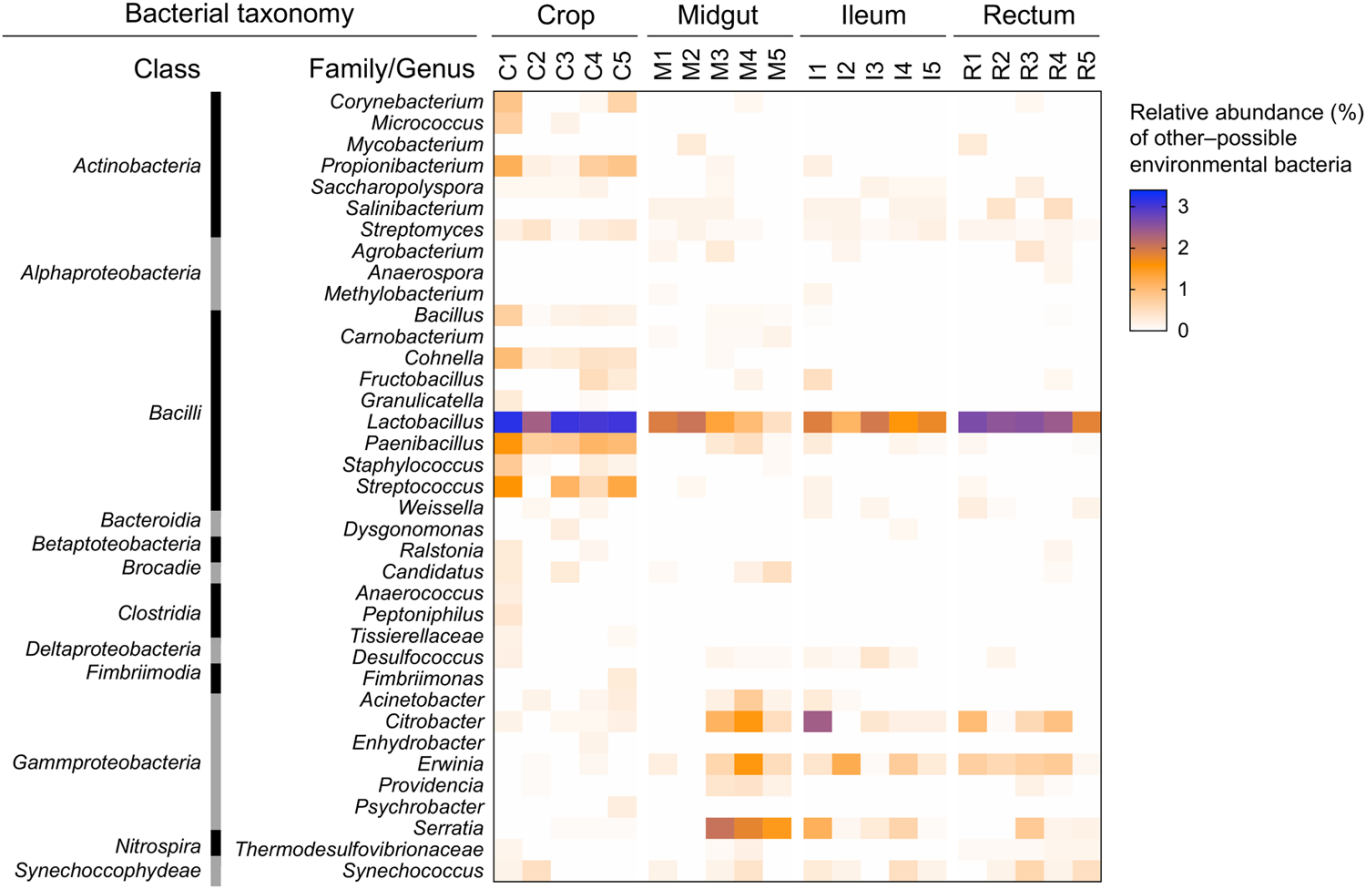
Distribution of other-possibly environmental bacteria along the honeybee gut compartments. Heat map represent the distribution of other-possibly environmental bacteria classes and families/genera detected from the analysis of the total bacterial communities (i.e., DNA; Fig. 2f) along the four gut compartments (the crop, midgut, ileum, and rectum); values are expressed as the relative abundance (%) of the normalized reads.

**Fig. 5:**
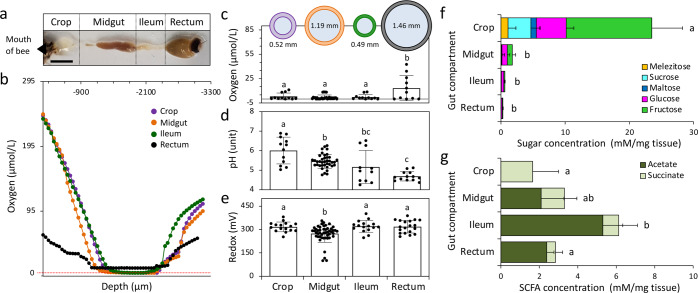
Physico-chemical and metabolite characterization of the honeybee gut compartments. **a** Honeybee gut compartments (the crop, midgut, ileum, and rectum) are depicted (bar = 0.5 cm). **b**–**e** Physico-chemical parameters measured by microsensors are reported for each gut compartment. **b** Representative radial profile of the oxygen concentration (μmol/l) along the gut compartments (i.e., the crop, midgut, ileum, and rectum) of honeybees. Depth refers to the distance covered by the electrode tip starting from the agarose surface (details in “Materials and methods”). **c** Schematic representation of the diameters of the gut compartments (circles at the top of the panel); diameters were expressed in mm, and their mean ± standard deviation (*n* = 12) are indicated by the internal and external circles, respectively. Details on the organ diameters are reported in Supplementary Table S12. The oxygen concentration measured in the central regions of the gut compartments (*n* = 11) are expressed as μmol/l. Microelectrode profiles of the (**d**) pH and (**e**) redox potential along the honeybee gut compartments (*n* = 12 and *n* = 16, respectively); values are expressed as unit and mV, respectively. Concentration (mM/mg of tissue) of (**f**) sugars and (**g**) short-chain fatty acids (SCFAs) in the gut compartments (*n* = 3); values are expressed as mean ± standard deviation. Letters show the results of Tukey’s multiple comparison test. Italian *A. mellifera ligustica* forager bees were considered for physico-chemical and metabolite analyses.

These dominant bacterial phylotypes co-existed alongside a high number (164 OTUs) of less-frequent bacterial OTUs (classified as other-possibly environmental bacteria, according to Kwong et al.^17^; see Supplementary Data 1). They were unevenly distributed along the gut compartments and were chiefly affiliated with *Gammaproteobacteria* (*Erwinia*, *Serratia*, *Citrobacter*, and *Acinetobacter*), *Bacilli* (*Streptococcus*, *Paenibacillus*, and *Lactobacillus* not belonging to Firm-4 or Firm-5), and *Actinobacteria* (*Propionibacterium* and *Streptomyces*; Table 1 and Fig. 4). These 164 other-possibly environmental bacterial OTUs were not amplified in the extraction blank and non‐template amplification controls (Supplementary Table S4), excluding their origin from laboratory work contaminations^45^. Although the acquisition of environmental bacteria may have been resulted from stochastic processes (e.g., nectar and pollen bacterial diversity^46,47^), their assembly was significantly affected by gut compartmentalization in terms of composition (i.e., niche partitioning; *F*_3,16_ = 4.4, *p* = 0.001; Figs. 2f and 4; *n* = 190, *p* < 0.0001, *R*^2^ = 0.14; Supplementary Fig. S3b) and richness (*F*_3,16_ = 4.7, *p* = 0.02; Supplementary Fig. S2c). Samples were distributed in a horseshoe shape ordination (arrow in Fig. 2f) in the space defined by the canonical analysis of principal coordinates (CAP), which indicated the presence of an ecological gradient. Moreover, this partitioning was confirmed by a higher contribution of OTUs turnover (i.e., species replacement^48^) to the beta-diversity pattern along the gut (Fig. 3b); specifically, environmental bacterial species tend to replace each other along the gut (Fig. 4), which is likely owing to the changing physico-chemical conditions of the gut (see Fig. 5 and its discussion) and biological correlations among members of the microbiome. For instance, the environmental bacterial species richness in the crop—the first compartment receiving environmental food source—was higher than that of the other gut portions (Supplementary Fig. S2c). In this compartment, environmental bacterial OTUs were unequally distributed across taxonomic groups (Supplementary Fig. S2d) and were dominated by *Lactobacillus*, *Paenibacillus*, *Bacillus*, *Streptococcus*, and *Propionibacterium*, the proportion of which was noted to be limited in other compartments (Fig. 4).

Our data regarding Italian *A. mellifera ligustica* (Fig. 2c, d, f) were confirmed by an analysis of *A. mellifera jemenitica* individuals collected in Saudi Arabia (Supplementary Fig. S5a, b and Supplementary Data 1). In this subspecies, we observed a significant compartmentalization of core and other-possibly environmental bacteria along the gut compartments (*F*_3,16_ = 2.69, *p* = 0.001 and *F*_3,16_ = 2.1, *p* = 0.009, respectively). Although the core bacteria followed the recognized trend of distribution and abundance (i.e., dominance of *Snodgrassella* and *Gilliamella* in the midgut and ileum and *Lactobacillus* Firm-4 and Firm-5 in the crop and rectum), other-possibly environmental bacteria showed a relative abundance ranging from 14%–17% in the initial portion of the gut to 4%–8% in the distal portion with a dominance of *Enterobacteriaceae* (*Serratia*, *Klebsiella*, and *Shigella*), *Morganellaceae* (*Morganella*, *Providencia*, and *Proteus*), *Orbaceae*, and *Rhizobiaceae* (Supplementary Data 1). Other-possibly environmental bacteria were also detected in Saudi Arabian nurse honeybees (Supplementary Table S5), following the same compartmentalization pattern observed for foragers (*F*_3,15_ = 3.54, *p* = 0.001; Supplementary Fig. S5e). Notably, the environmental bacteria detected in Saudi Arabian honeybees exhibited a markedly different taxonomic composition respect to the one in Italian honeybees, emphasizing the environmental origin of these members of the microbiome.

Because the culture-independent methods used in the present study cannot discriminate between non-viable dead bacterial cells (i.e., DNA from lysed bacterial cells) and live/active bacterial cells, we analyzed the cDNA synthetized from total RNA of active members of the bacterial community. Amplification of the 16S rRNA gene from cDNA confirmed that other-possibly environmental bacteria were present (relative abundance of 2%–6%) and metabolically active within the gut microbiome (Fig. 2e, Supplementary Fig. S6 and Supplementary Data 1). The abundance of these bacteria along the gut ranged from 2% to 8% (DNA), of which 2%–6% (cDNA) was metabolically active. The observed difference may occur owing to a part of such bacteria being filtered/selected by the gut environment as well as biotic interactions within the honeybee gut (i.e., anoxia and subacid pH), whereas the remaining bacteria are unable to survive and were thus detected as environmental DNA (∼2%). Their possible functional role was inferred from taxonomy by using the Tax4Fun2 platform (Supplementary Data 2). These bacteria were involved in (1) secondary metabolite biosynthesis, such as antibiotic synthesis, which can defend the insect host against parasites^49,50^ and/or regulate the microbiome balance via competitive interactions among members of the gut microbiome^51,52^; (2) xenobiotic biodegradation, which may aid the honeybee with detoxification of external substances from the diet^53^; and (3) glycan biosynthesis and degradation, which reveals their association with the gut and interaction with the host^54,55^. Furthermore, we observed that other-possibly environmental bacteria contribute to the metabolism of carbohydrates, amino acids, and lipids as well as the biosynthesis of cofactors and vitamins (Supplementary Data 2), thereby suggesting their ancillary contribution beyond that exerted by the core bacteria in degrading host-ingested substrates and distributing essential nutrients^3,56^.

An association between gut and environmental bacteria is evident in insects^57^, including honeybees^5,17,29,43^, but the magnitude and contribution to host function, performance, and/or health remains unelucidated. In the present study, adult forager honeybees were collected from the front of the hive following exposure to the natural environment (i.e., flowered prairie), thereby enabling the recruitment of main core bacteria from the hive and contact among mates and environmental bacteria during harvest^3,29,44^. Such recruitment processes are affected by the surrounding environment and pollination landscape (e.g., microbiome variation in nectar, pollen, and hive materials^46,58^), resulting in strong variation among the frequencies and biodiversity of bacteria associated with honeybees^5,21,29,43^. Although we anticipated that the type of environmental bacteria (non-gut bacteria, such as *Actinomycetales*, *Alphaproteobacteria*, *Enterobacteriaceae*, *Pseudomonadales*, Firmicutes, and *Xanthomonadaceae*^43,58^) would vary depending on environmental conditions (such as location, season, food source, and climate), their recruitment and distribution was determined by the interaction between the physico-chemical conditions and biological network of the honeybee gut. For instance, Kešnerová et al.^5^ found that minor community members (i.e., other-possibly environmental bacteria) detected in the short-lived forager and nurse honeybees (∼4 weeks) were present in lower concentrations in long-lived winter bees (~6 months) and can be transient colonizers that are mainly acquired from the surrounding environment during foraging. Winter bees feed strictly on food stored in the hive (i.e., aged pollen) and retain their feces all winter, affecting the physico-chemical conditions and the availability of nutrients in the gut, and consequently impacting the ecology of their gut microbiota^5^; despite it was quantified that winter bees had the highest bacterial loads, they were characterized by a lowest bacterial community alpha diversity, with dominance of *Bartonella* and *Commensalibacter*, reduction of opportunistic colonizers (*Apibacter*, *Bombella*, or *L. kunkeii*) and environmental bacteria (such as, *Serratia* and *Klebsiella*) possibly by the specific dietary conditions of these bees and the activation of host resistance mechanisms in place to favor their and overall colony survival^5^. Nevertheless, several studies have shown that non-core environmental bacteria can provide benefits: bacterial strains isolated from honeybee gut belonging to *Lactobacillus kunkeei* and *Bacillus* sp. were found to protect the host from pathogens such as *Paenibacillus larvae* and *Melissococcus plutonius*^12,59^, whereas *Staphylococcaceae* and *Enterobacteriaceae* members were able to metabolize plant derivates and sugars that are toxic to the host^4^. In natural environmental systems, members of the rare prokaryotic biosphere may represent a substantial source of genomic and functional features^60^. Similar to the importance of low-abundant bacteria in re-shaping ecosystems, they can enhance host immunity or prevent pathogen colonization in plant and animal associations^60,61^. Although generalist broad metabolic processes, such as respiration, metabolic potential, and cell yield are performed by dominant phylotypes, more specialized functions, such as the degradation of specific compounds, can be performed by low-abundant, niche-specialist phylotypes^62^. Currently, technical limitations preclude the ability to investigate the functioning of the in vivo ecosystem functioning of the rare biosphere in the gut interactome. For instance, absolute germ-free adult honeybee individuals do not exist and thus cannot be studied. However, published studies have reported a significant reduction in the total amount of bacterial cells in honeybee guts (i.e., < 10^5^ bacterial cells per gut consisting of an erratic mix of bacterial species^1,39^) but never complete elimination, rendering this approach unsuitable for defining whether rare/environmental microbial components contribute to the maintenance of microbiota homeostasis in the honeybee gut.

Future investigations into other-possibly environmental bacteria, often masked by dominant bacterial phylotypes, will further elucidate microbial community recruitment, assembly, and functioning in the honeybee gut.

### Compartmentalization of the mycobiota along the honeybee gut

The honeybee gut hosts microbial cells that are larger than bacterial cells, such as those of yeasts and other fungi (Fig. 1e, f and Supplementary Fig. S7). The number of fungi was consistently lower than that of bacteria; however, their abundance followed the same trend, with a progressive increment observed from the crop to the rectum (4.9 × 10^2^, 5.8 × 10^2^, 1.7 × 10^3^, and 2.9 × 10^3^ fungal cells in the crop, midgut, ileum, and rectum, respectively; data normalized according to Lofgren et al.^63^; Fig. 2b and Supplementary Table S1). Cultivation-dependent approaches were applied to three different honeybee species (i.e., *A. mellifera ligustica*, *A. mellifera jemenitica*, and *A. florea*) from beehives in five locations in two different countries (Italy and Saudi Arabia; Supplementary Table S6), which confirmed the presence and viability of the fungal cells (Table 2). The mean number of fungal colony-forming units (CFUs) isolated from the anterior (the crop and midgut) and posterior (the ileum and rectum) parts ranged from 2.7 × 10^2^ to 1 × 10^4^—consistent with quantitative polymerase chain reaction (qPCR) findings (note the single compartments reported in Fig. 2b). Among these isolates (90 in total) the most abundant genera retrieved were *Starmerella* (31%), *Hanseniaspora* (12%), *Aspergillus*, and *Naganishia* (both 11%); the genera *Aureobasidium*, *Moniliella, Candida*, and *Penicillium* were less abundant (Supplementary Table S7). *Starmerella* yeasts isolated from Italian honeybees (*A. mellifera ligustica*) clustered with the strains *S. bombicola*, whereas in the Saudi Arabian honeybees (*A. mellifera jemenitica* and *A. florea*), these isolates mainly clustered with *Starmerella meliponinorum* (Supplementary Fig. S8). All isolates of *Hanseniaspora* strains were identified as close relatives of *H. uvarum* (Supplementary Fig. S8). Using the size, shape, and morphology of *Starmerella bombicola* and *Hanseniaspora uvarum* isolates as a reference (scanning electron microscopy (SEM) micrographs, Fig. 1g, h and Supplementary Fig. S9, similar round- and club-shaped yeast cells were identified in the honeybee gut (Fig. 1e, f and Supplementary Fig. S7a–c). Moreover, other yeast-like and fungal-like morphologies were detected (Supplementary Fig. S7d–f).

**Table 2:** Abundance of culturable fungi associated with two gut portions: anterior (i.e., the crop and midgut) and posterior (i.e., the ileum and rectum).

Honeybee species	Location	Gut region	PDA (CFU per anterior organs)		YM (CFU per posterior organs)	
			Mean	SD	Mean	SD
*A. mellifera ligustica*	Milan	Anterior	1.01 × 10^4^	5.34 × 10^3^	3.37 × 10^3^	1.78 × 10^3^
		Posterior	9.50 × 10^3^	5.25 × 10^3^	3.17 × 10^3^	1.75 × 10^3^
*A. mellifera jemenitica*	Jeddah	Anterior	8.00 × 10^2^	6.93 × 10^2^	8.00 × 10^2^	4.00 × 10^2^
		Posterior	1.47 × 10^3^	1.22 × 10^3^	1.07 × 10^3^	8.33 × 10^2^
	Makkah	Anterior	2.67 × 10^2^	1.15 × 10^2^	3.00 × 10^2^	1.15 × 10^2^
		Posterior	1.40 × 10^3^	5.77 × 10^2^	3.27 × 10^3^	2.94 × 10^3^
	Madinah	Anterior	4.76 × 10^3^	4.05 × 10^3^	7.64 × 10^3^	7.88 × 10^3^
		Posterior	8.67 × 10^2^	1.15 × 10^3^	2.36 × 10^3^	1.91 × 10^3^
*Apis florea*	Thuwal	Anterior	1.20 × 10^3^	9.38 × 10^2^	7.50 × 10^2^	8.39 × 10^2^
		Posterior	2.95 × 10^3^	2.88 × 10^3^	3.53 × 10^3^	5.77 × 10^3^
Total mean		Anterior	3.43 × 10^3^	4.13 × 10^3^	2.57 × 10^3^	3.08 × 10^3^
		Posterior	3.24 × 10^3^	3.59 × 10^3^	2.68 × 10^3^	1.03 × 10^3^

Values are expressed as mean ± standard deviation (SD) of colony-forming unit (CFU) per gram of gut portion. Mean of all anterior and posterior compartments is also reported. The origin of individuals is specified in the table.

*PDA* potato dextrose agar, *YM* yeast medium.

An analysis of the fungal community yielded 118 OTUs (Supplementary Data 1). The crop hosted a fungal community that was significantly different from the following compartments of the gut (Fig. 2g and Supplementary Table S2), with a higher Shannon diversity but similar richness (Supplementary Fig. S2e, f). Similarity among communities significantly decreased from the crop to the rectum (*n* = 163, *p* < 0.0001, *R*^2^ = 0.34; Supplementary Fig. S3c), suggesting a niche partitioning and compartment specificity. Moreover, the beta-diversity pattern of the fungal community, similar to that of other-possibly environmental bacteria along the gut, was mainly explained by variations in species composition^48^ (species turnover; Fig. 3c), suggesting the presence of a well-established gradient along the gut. The genera *Hanseniaspora* (35%)*, Penicillium* (24%), *Cladosporium* (9%), *Malassezia* (7%), *Starmerella* (5%), and *Candida* (2%) composed the crop mycobiota (Fig. 2h; data normalized according to Lofgren et al.^63^). Among these, *Cladosporium, Penicillium, Candida*, and *Malassezia* significantly reduced in abundance along the intestinal trait, thereby providing space for the proliferation of *Saccharomycetales* yeasts (*Hanseniaspora* and *Starmerella*), which subsequently become dominant (86%, 98%, and 91% relative abundance in the midgut, ileum, and rectum, respectively). The other detected fungi (*Cladosporium, Penicillium*, *Malassezia*, and *Candida*, among others; Fig. 2h, Supplementary Fig. S8, Supplementary Data 1, and Supplementary Table S7) were ubiquitous species found in varied habitats (including soil) as well as common plant pathogens and endophytes^64^ and animal associates^65^. Therefore, we consider fungi as microbiome members of environmental origin that were acquired by honeybees during food intake, particularly during foraging^34,38^. This was further confirmed by the fact that *A. mellifera jemenitica* forager honeybees from Saudi Arabia (Madinah) were colonized by complex communities dominated by *Zygosaccharomyces*—yeasts belonging to the *Saccharomycetaceae* family, which are often detected in hive material and bee guts^34,38,66^—along with members of *Alternaria*, *Aspergillus*, *Eremothecium*, and *Fusarium* (Supplementary Data 1). Notably, mycobiota members exhibited differential distribution across the gut compartments (*F*_3,16_ = 2.11, *p* = 0.01; Supplementary Fig. S5), confirming the trend that was previously observed in Italian honeybees (Fig. 2g). The presence of fungi, as gut-associated microorganisms, was also detected in nurse honeybee gut (Saudi Arabia) with a spatial-differentiation across the gut compartments (*F*_3,16_ = 3.2, *p* = 0.002). The mycobiota of nurse and forager honeybees from Saudi Arabia was dominated by the same main members belonging to *Zygosaccharomyces* (Supplementary Table S5 and Supplementary Data 1); it was not present in the Italian honeybees, presumably owing to the different surrounding environment (e.g., vegetation) of the sampling sites (Italy and Saudi Arabia). However, despite the elevated diversity of the mycobiota along the entire gut^34,38^, the dominant members of the mycobiota in the honeybees analyzed were consistently fermentative yeasts (*Hanseniaspora* and *Starmerella* in Italy, and *Zygosaccharomyces* in Saudi Arabia); based on their taxonomy and metabolic information available in literature, they can have a role in food digestion (i.e., sugar fermentation and nutrient recycling) and favor trophic and spatial interplay among gut microorganisms^67–69^ (Supplementary Data 2).

Fungal members have previously been detected in the honeybee gut, with their origin being mainly ascribed to food intake^32–34,37,38,70^. Yeasts are common gut inhabitants of insects, such as *Drosophila* fruit flies, wasps, and mosquitoes^12,71–74^, which may be owing to their ability to thrive in sugar-rich environments^75^. In previous metagenomics surveys of honeybee-associated microbes, sequences related to *Saccharomycetales* were retrieved among fungal components^33–35,76,77^. Indeed, species of the *Starmerella* and *Hanseniaspora* genera, along with those of the *Saccharomyces*, *Candida*, and *Zygosaccharomyces* genera, have been isolated from flowers, fruits, and flower-visiting insects^72,78^, including bees^34,38,79^. Therefore, we hypothesized that such yeasts are acquired by honeybees via their diet, from the surrounding environment, or mates, which is similar to what was observed for low-abundant bacteria^17^. Moreover, the dominance of *Saccharomycetales* yeasts in our foragers—i.e., *Hanseniaspora* and *Starmerella* in Italian bees and *Zygosaccharomyces* in Saudi Arabian bees (Supplementary Data 1)—was justified by the favorable niche that the honeybee gut provides for such sugar-fermentative microorganisms^34,66,68^. The frequent association between fungi (i.e., yeast-like) and the insect gut suggests that they are prominent in host homeostasis, physiology^68^, and development^80^, which possibly provides essential nutritional support, including enzymes, essential amino acids, vitamins, and sterols^68^. Conversely, yeasts benefit from the protected environment of the gut and a dispersion service provided by the insect host^74,81^. For example, *S. cerevisiae* spores from tetrads increase the outbreeding of the yeast^82^. Furthermore, yeasts such as *Starmerella bacillaris* are unable to grow in vitamin-free media, which is suggestive of their dependence on other gut inhabitants^83^.

In the gut, the fungal cells were consistently surrounded by bacteria (e.g., *Starmerella* and *Hanseniaspora* in Fig. 1e, f); this physical contact can establish interactions among gut-associated microorganisms and favor a trophic and spatial interplay among them (intra group) as well as with the core and non-core bacterial components. For example, in an artificial microbial community, the nitrogen overflow of *S. cerevisiae* reportedly enabled the survival of two *Lactobacillus* species^84^. This likely occurs in the honeybee gut microenvironment, where yeasts and *Lactobacillus* strains are both present in the same compartments. Moreover, considering the cell size (e.g., in Fig. 1e–h), fungal cells may model the spatial interactions and metabolic flow within the gut microbial community by designing the architecture of the gut and influencing the geometry of interactions among bacterial components—bacteria might exploit the surfaces made available by fungal cells to establish ecological interactions among them^67,85^.

### Physico-chemical conditions along the honeybee gut compartments

Complex physico-chemical conditions characterize the insect gut ecosystem^86^. Compared with a previous study that focused on conventional honeybees with a reconstituted gut microbiota under laboratory conditions (sterile diet accompanied by hindgut homogenate of nurses)^39^, in the present study, we ascertained these conditions along the intestinal tract of forager honeybees (Fig. 5a and Supplementary Table S9), which were collected in their natural environment. Suboxic zones with positive redox potentials and subacid pH were confirmed along the entire honeybee digestive tract (Fig. 5 and Supplementary Fig. S10). The partial oxygen pressure (pO_2_) measured in the center of the gut lumen reached values that were close to zero (< 5 μmol/l) in the crop, midgut, and ileum, whereas they flattened to a significantly higher concentration in the rectum (range, 0–37.5 μmol/l; 0%–13% air saturation, and 0%–2.8% oxygen; *F*_3,61_ = 7.769, *p* = 0.0002; Fig. 5b, c; Supplementary Table S8). The micro-profiles revealed a significant decrement of subacid pH values along the digestive tract from the proximal to distal gut compartments (the crop: 6.1 ± 0.2, midgut: 5.6 ± 0.1, ileum: 5.3 ± 0.9, rectum: 4.8 ± 0.2; *F*_3,70_ = 15.4, *p* < 0.0001; Fig. 5d and Supplementary Fig. S11). This progressive reduction was significantly correlated with the increment of the microbial load detected by qPCR (Fig. 2b; Pearson’s product-moment correlation; *t* = −2.36, df = 18, *p* = 0.03; *ρ* = −0.49; Supplementary Fig. S11). Finally, although the gut was anoxic/strongly hypoxic, positive values of the redox potential were consistently detected along the gut (range, 215–370 mV; crop: 292 ± 7, midgut: 266 ± 4, ileum: 296 ± 9, rectum: 293 ± 8), with the midgut showing the lowest significant values (Fig. 5e and Supplementary Fig. S10). Based on these results, we confirmed that the four physical portions of the gut differed in size and shape (representation in Fig. 2a) as well as in their physico-chemical milieu (PERMANOVA: *F*_3,61_ = 10.756, *p* = 0.001; pairwise comparison in Supplementary Table S9a). Among these variables, the pH significantly explained core and other-possibly environmental bacterial, but not fungal, variations (Supplementary Table S10). Among the core bacteria, pH values controlled the abundance of *Bartonella* (OTU_3) and *Bifidobacterium* (OTU_2; Supplementary Fig. S12) with a higher relative abundance under the lower pH conditions that were created by the fermentative metabolisms of the same bacteria^56,87,88^. Conversely, the other-possibly environmental bacteria *Paenibacillus* (OTU_20), *Propionibacterium* (OTU_28), *Cohnella* (OTU_29), and SAR406 (OTU_21) were positively correlated with higher pH values within the crop (Supplementary Fig. S13), which is consistent with their pH optima near neutral conditions^89–92^.

The co-presence of anoxic conditions with positive redox potentials was observed in the intestinal tract of other arthropods and in honeybees^39,86,93^, and was caused by the production of oxidized products of the microbial metabolic activities during fermentation^8^. The creation of an anoxic/strongly hypoxic condition was mainly ascribed to the oxygen scavenger activity of gut wall-associated microorganisms, such as the obligate aerobe *S. alvi*^94^ (mainly detected in the ileum^39^) and acetic bacteria (*Commensalibacter* and *Bombella*; mainly found in the crop and midgut). Notably, *Snodgrassella*, a major oxygen scavenger, could be located in other gut compartments^42,95^; in our honeybees, it was consistently detected in the midgut, ileum, and rectum (Fig. 2b). Contrary to the anoxic condition detected by Zheng et al.^39^, a variable percentage of oxygen was observed in the rectum of the forager honeybees (Supplementary Table S8); this could be ascribed to the intrinsic dynamics of this gut compartment, in which different levels of pollen content, dietary habits, waste accumulation, fermentation processes, and microbial load can occur^5,14,39^, particularly for forager honeybees, which under field conditions supposedly consume less pollen and more honey and nectar compared with nurses. Such oxygen depletion creates a favorable environment for facultative anaerobes and microaerophilic fermentative bacteria (i.e., *Gilliamella*, *Lactobacillus* spp., and *Bifidobacteium*^1,4,39^) and anaerobic microorganisms inhabiting digestive tracts (e.g., coliforms, staphylococci, *Bacillus* spp.)^96^. The establishment and activity of these bacteria can determine a significant change in the pH^39^, as was observed in our study. Indeed, in vitro metabolic characterization showed the way in which strains isolated from the honeybee gut significantly reduced the pH of the cultural media owing to the release of acetic and lactic acids during fermentative processes^26,39,94,97^, thereby confirming the correlation between the bacterial load and pH reduction. Despite the consistent positive redox observed in the honeybee gut, overall higher values were measured in field-foraging honeybees compared to laboratory honeybees possessing a reconstituted microbiome^39^ (Supplementary Table S8).

### Metabolic products along the honeybee gut compartments

Genomic and transcriptomic analyses, along with in vitro experiments, have shown that the predominant metabolic activity in the honeybee gut was the microbial fermentation of sugars and complex carbohydrates (e.g., pectin) into organic acids (among others^4,8,9,39^). In the present study, we evaluated the concentration of dietary metabolites (sugars) and their fermentation products (short-chain fatty acids [SCFAs]) along the gut compartments. Quantification of the metabolite milieu (SCFAs and sugars) along the intestinal tract confirmed the presence of a spatially explicit distribution pattern that contributed to gut compartmentalization^25^ (PERMANOVA: *F*_3,8_ = 15.123, *p* = 0.001; Supplementary Table S11; pairwise comparison in Supplementary Table S9b). In particular, a significant decrease was observed in sugar concentration along the gut (*F*_3,8_ = 14.35, *p* = 0.0014), with the main reduction occurring in fructose (from 13.32 ± 4.8 to 0.1 ± 0.06 mM/mg tissue in crop and rectum, respectively) and glucose (from 4.7 ± 1.2 to 0.2 ± 0.08 mM/mg tissue) concentrations as well as in the total consumption of sucrose, melezitose, and maltose from the crop to the ileum (Fig. 5f and Supplementary Table S11); maltotriose remained within the detection limit in all compartments. Notably, among the sugars, sucrose resulted in a significant variable that controlled the microbiome assembly (core bacteria, other-possibly environmental bacteria, and fungi) across the gut compartments (Supplementary Table S10). It negatively influenced the relative abundance of *Bifidobacterium* (OTU_2), *Frischella* (OTU_9), and *Snodgrassella* (OTU_4) members of the core phylotypes (Supplementary Fig. S12), which was consistent with their preferential localization in distal parts of the honeybee gut (the ileum and rectum; Fig. 2d), where the sucrose concentration was low (Fig. 5f); conversely, *Lactobacillus* Firm-5 (OTU_120) showed an opposite trend (Supplementary Fig. S12), which was expected based on its metabolic capability to utilize fructose derived from sucrose^98^. Furthermore, we detected a total of eight other-possibly bacterial OTUs associated with the presence of sucrose (Supplementary Fig. S13). These OTUs belonged to the *Enterobacteriaceae* family, and other *Lactobacillus* spp., showed both positive (OTU_10 and OTU_17) and negative (OTU_20, OTU_29, OTU_108, OTU_118, OTU_174, and OTU_258) relationships with this variable. Along with these results, sucrose positively influenced the presence of fungal members, such as *Penicillium* (OTU_41 and OTU_575), *Ganoderma* (OTU_168), and unclassified fungi (OTU_3 and OTU_29), which had higher relative abundance in the crop, whereas the fermentative yeast *Hanseniaspora* sp. (OTU_739) showed an increased abundance with the decrease of sucrose (Supplementary Fig. S14).

The two monosaccharides glucose and fructose and the disaccharide sucrose are the most common sugars of nectar and honey, which together with pollen, constitute the major components of the forager honeybee diet^99,100^. These simple sugars are, in part, passively absorbed via the host midgut wall^101^. Accordingly, these sugars were mainly detected in the crop, which was the first intestinal compartment following the honeybee mouth, and drastically reduced in the midgut. Unlike laboratory conditions, in a natural ecosystem, forager honeybees load their crop with a certain amount of honey before leaving the hive to sustain their flight energy cost^102^. The amount of honey loaded is adjusted according to several factors, such as distance and food source type that they intend to collect and carry to the hive^102^, which explain the high variability observed in terms of sugar concentration detected in the guts of these honeybees. In the absence or limited availability of nectar, honeybees can forage on honeydew (i.e., hemipteran waste product from plant phloem). In addition to nectar sugars, honeydew contains disaccharides (e.g., maltose) and trisaccharides (e.g., melezitose)^103^, which were also detected with high variability in forager honeybee guts in the present study (Fig. 5f). The type of sugar affects the relative abundance of dominant bacterial members in the microbiota of honeybee guts^23,40^, consequently leading to an alteration of the gut metabolic and physico-chemical conditions^1,4,14,39^, which was confirmed by comparing field-foragers bees with laboratory-reared bees (Supplementary Table S8).

Following consumption, the portion of sugars that is not directly assimilated by the host is further metabolized by members of the gut microbiome^4,7,15,23^, as clearly shown by Zheng et al., who used honeybees with a reduced bacterial load (< 10^5^ bacterial cells per gut)^39^. As expected, the decrease in sugar concentration observed along the gut compartments (Fig. 5f) was paralleled by a gradual increase in organic acids and SCFAs (*F*_3,8_ = 7.44, *p* = 0.011; Fig. 5g, Supplementary Tables S11, and Supplementary Fig. S11). For instance, when acetic acid reached its highest concentration in the ileum, the high variability of succinic acid did not determine consistent trends; on the contrary, all the other organic acids and SCFAs that were measured (lactic, propionic, malic, formic, and butyric acids) were within the detection limit (< 0.01 mg/ml). SCFA accumulation in the honeybee ileum, followed by the rectum and midgut, can be ascribed to the higher bacterial load hosted by these compartments compared with the crop (Fig. 2b and Supplementary Fig. S11). In addition, we observed that among the SCFAs that were analyzed, acetic acid significantly affected the fungal distribution along the intestinal tract compartments (Supplementary Table S10). Members of the *Penicillium* genus (OTU_41, OTU_575, and OTU_458) and one unclassified fungus (OTU_20) were negatively correlated with the acetic acid concentration detected (Supplementary Fig. S14), which was possibly owing to the fact that organic acid, such as acetic acid, possessed antifungal activities^104–106^; it should be noted that we did not find any SCFAs that influenced the core or other-possibly environmental bacterial distributions (Supplementary Table S10).

Recent metagenomic, metatranscriptomic, metabolomic, and in vitro analyses have revealed that the breakdown and fermentation of host dietary macromolecules into alcohols, SCFAs, and other organic acids—useful to sustain host growth and development—are the predominant metabolic functions of the core gut microbiome of honeybees^1,4,107^. For instance, the production of SCFAs was mainly ascribed to the metabolic activity of the *Lactobacillus* Firm-5 clade (monocolonization, gnotobiotic, and reconstructed gut microbiota experiments^1,39^) and the digestion of pollen-derived compounds was mediated by *Gilliamella* and *Bifidobacterium* (e.g., pectin and hemicellulose)^4,7–9,39^. Notably, the presence of organic acids (e.g., succinate) in the crop—where these bacteria were limited (Fig. 2b)—can be ascribed to the fact that the honey load carried by foragers before leaving the hive^102^ naturally contains these molecules^108^.

Genes for sugar transport and carbohydrate-degrading enzymes for breaking down nectar sugars (and other potentially toxic carbohydrate) are specifically enriched in the *Gilliamella*, *Bifidobacterium*, and *Lactobacillus* Firm-4 and Firm-5 bacterial clades^4,109^. An in vitro study showed that bacterial isolates belonging to the *Gilliamella*, *Lactobacillus* Firm-5, and *Bifidobacterium* clades were associated with transcribing the genes encoding for acetate kinase and L-lactate dehydrogenase, thereby being responsible for acetic and lactic acid production, respectively, particularly in the ileum and rectum compartments^4^. Despite their capacity, genomic diversity and metabolic variations within each clade were observed, suggesting that each core microbiome member differentially contributed to honeybee metabolism^4,18,26^. Conversely, these fermentative metabolisms of main nectar sugars (glucose, fructose, and sucrose) were not consistently detected in isolates classified as non-core gut members (other-possibly environmental bacteria)^4^. In addition, more distantly related bacteria not belonging to the honeybee core (*Staphylococcaceae*, other *Lactobacillales*, and *Enterobacteriaceae*) could not utilize pollen-derived compounds, such as pectin, but could contribute to organic acid production by metabolizing secondary sugars that are common in the honeybee diet (mannose, rhamnose, and galactose)^4^.

Reduction of the gut bacterial load in laboratory-reared honeybees unequivocally revealed that bees with a depleted-microbiome (i.e., < 10^5^ bacteria per gut consisting of an erratic mix of bacterial species) exhibited less accumulation of fermentative products (SCFAs) in the ileum and rectum gut portions with a significant reduction in body weight^39^. Interestingly, using the gut of honeybees monocolonized by individual bacterial strains of the corbiculate core, it was possible to demonstrate that most of the metabolic output of the bee gut microbiota can be ascribed to the metabolic activities of single microbial community members^1^. For instance, succinic acid was exclusively produced in honeybees colonized by *Lactobacillus* Firm-5 strains^1^, which possibly explain the presence of this metabolite in the midgut of honeybees studied in the present work where Firm-5 were also present.

Considering the increasing evidence regarding the effect of diet and environment on the microbial composition and metabolic output of the honeybee gut^1,5,14,40^, it is reasonable to deduce that the differences observed in sugars and SCFA concentrations between our study and that of Zheng et al.^39^ were likely owing to the different diet and environmental conditions to which the bees were subjected.

## Conclusion

In the present study, we demonstrated that compartmentalization of the gut microbiota in honeybee occurs for main bacterial phylotypes as well as for non-core, less-prevalent environmental bacterial and fungal members. We comprehensively determined their distribution, diversity, and assembly for all four gut compartments. Compartmentalization of these microbial groups indicates that similar to the main bacterial phylotypes, they have an explicit pattern of distribution along the different conditions of the gut and are not simply acquired via diet and passively transited along the gut. Our results can provide the basis for further investigations regarding the ways in which fungi and environmental bacteria interact with the core microbiome to shed light on additional mechanisms that contribute to gut homeostasis. Elucidating the dynamics of the gut microbiome is crucial for understanding, explaining, and predicting the degree of host resilience while experiencing changes in environmental conditions and challenges by stressors and pathogens.

## Methods

### Honeybee sampling

Adult forager *Apis mellifera ligustica* (Hymenoptera: Apidae) bees (aged > 21 days) were collected from the front of experimental apiaries located at the Department of Agricultural, Forest and Food Sciences (DISAFA, Grugliasco, Torino, Italy) following exposure to the natural surrounding environment between September 2016 and October 2018 (Supplementary Table S6). Additionally, between May 2019 and January 2020, adult forager bees (*A. mellifera jemenitica* and *A. florea*) were obtained from sites across Saudi Arabia (Medina, Thuwal, Jeddah and Makkah; Supplementary Table S6). All collected specimens were immediately transported to the laboratory and processed. Animals used in our experiments were maintained and treated in compliance with the guidelines specified by the Italian Ministry of Forestry and Agriculture. In addition, all necessary permits were obtained when the research was conducted, and all experiments and procedures were approved by the University of Torino. The research at King Abdullah University of Science and Technology (KAUST) was performed in compliance with Saudi Arabian and International guidelines.

### Honeybee gut dissections

Forager honeybees were chilled in a sterile Petri dish at 4 °C for 10 min, following which they were individually transferred to 10 ml sterile plastic tubes. They were surface sterilized by washing the individuals with 1% sodium hypochlorite and then 97% ethanol. Specimens were then rinsed three times with sterile water before dissection and extraction of the gut. For DNA-based and metabolite analysis, the whole intestine (attached to the thorax and the head of the insect; Fig. 5a) of a subset of samples (*n* = 50, *A. mellifera ligustica* from Italy and *n* = 50, *A. mellifera jemenitica* from Saudi Arabia, Supplementary Table S6) was extracted from the insect body, placed in a Petri dish, and frozen at –20 °C to avoid the release of internal gut content during dissection. Separation of the frozen guts was performed—starting from the crop, proceeding with the midgut, and concluding with separation of the ileum and rectum. Gut dissection was performed in Ringer’s solution under a stereomicroscope in sterile conditions using sterile forceps and needles, and the scalpel was sterilized between every cut. Specimens were discarded when any portion of the gut was released into the intestinal liquid during dissection of the fresh tissues or separation of the frozen tissues. The four gut compartments (i.e., the crop, midgut, ileum, and rectum) obtained were pooled (*n* = 10) in five separate tubes to obtain sufficient microbial DNA for molecular analysis. The pooled gut compartments were weighed using an analytical balance (Supplementary Table S12) and frozen at –80 °C for DNA and metabolite preservation.

### Fungal counts and isolation from honeybee adult gut

For the fungal isolation procedures, samples collected from Italy (*n* = 9, *A. mellifera ligustica*) and Saudi Arabia (*n* = 15, *A. mellifera jemenitica* and *A. florea*) were used (Supplementary Table S6). In this case, following surface sterilization, the honeybee gut was divided into two parts: the anterior part, including the crop and midgut, and the hindgut region, including the ileum and rectum. The obtained portions were transferred to a tube containing 0.9% NaCl (400 and 900 µl for single and pool of gut portions, respectively) and were immediately homogenized. A serial dilution was prepared and plated on yeast mold (YM) agar (Conda, Milano, Italy) and potato dextrose (PD) agar (Conda, Milano, Italy) supplemented with 100 µg/ml chloramphenicol to inhibit bacterial growth. Plates were incubated at 30 °C for 48 h. The colonies were counted, and the total CFU/mg of gut portions were determined. A total of 90 colonies were randomly selected and purified in new plates. The fungal strains were further inoculated in liquid media (YM and PD); the pure cultures thus obtained were used to extract DNA by boiling lysis to prepare the glycerinates with 20% glycerol, which was stored at –80 °C. Internal transcribed spacer 1 (ITS1) fragment was amplified using primers ITS1f and 5.8s^71^. Amplicons were sequenced and then aligned against the NCBI database using the BLAST tool. Sequences were deposited into the European Nucleotide Archive under accession numbers LR746502-LR746517 and LR798000-LR798072.

### Electron microscopy

All gut specimens used for SEM were dissected from fresh *A. mellifera jemenitica* obtained from Makkah and Medina regions of Saudi Arabia following surface sterilization. The whole guts dissected were fixed in a solution of 2.5% (v/v) glutaraldehyde in 0.1 M sodium cacodylate buffer (pH 7.2) and stored at 4 °C. Additional steps for the sample preparation were reported in Supplementary Method S1. The honeybee gut was visualized using transmission electron microscopy (Supplementary Method S2). The yeasts (*Hanseniaspora uvarum* L18 and *Starmerella bombicola* L28; Supplementary Table S7) that were isolated from the guts of Italian *A. mellifera ligustica* forager honeybees were grown in 5 ml PD broth media for 24 h at 30 °C on a rotary shaker at 100 rpm. Yeast cells were collected by centrifugation at 800 rpm for 10 min and fixed as described in Supplementary Method S3. All coated samples (guts and yeast strains) were visualized using the SEM Quanta 600 FEI at the KAUST Imaging and Characterization Core Lab.

### Extraction of nucleic acids

Overall, five pools of ten dissected gut compartments (a total of 50 crop, midgut, ileum, and rectum sections) were obtained from Italian and Saudi forager bees (*A. mellifera ligustica* and *A. mellifera jemenitica*, respectively; Supplementary Table S6); the compartments were homogenized in sterile 0.5 ml 1× TE (10 mM Tris-HCl pH 8, 1 mM EDTA pH 8) using sterile pestles for 1 min. After three freeze/thaw cycles (–80 °C for 10 min and 70 °C for 10 min), lysozyme (Sigma-Aldrich, St. Louis, MO, USA) was added to a final concentration of 0.5 mg/ml per sample, followed by incubation at 37 °C for 30 min. The obtained homogenizations were used for DNA extraction according to the protocol of sodium dodecyl sulfate–proteinase K-cethyltrimethyl ammonium bromide treatment by Vacchini et al.^110^. DNA was eluted in 1× TE [100 μl (midgut and rectum) or 50 μl (crop and ileum)] and stored at –20 °C. Further, sterile water was used as the control for the DNA extraction procedures to assess the presence of reagent contamination. The extracted DNA was used as the control in all the further molecular analyses. The concentration of DNA samples was measured using the Nanodrop ND-1000 spectrophotometer. The total RNA was extracted from single fresh gut sample of six forager bees (*A. mellifera jemenitica*) collected from Madinah (Saudi Arabia; Supplementary Table S6) and reverse transcribed to cDNA (Supplementary Method S4) to detect the transcriptionally active microbial cells that are associated with the gut microbiota.

### Abundance of bacterial and fungal communities by qPCR

qPCRs were performed using the CFX Connect™ Real-Time PCR Detection System (Bio-Rad). All bacteria obtained from *A. mellifera ligustica* (Italy) were quantified by evaluating the 16S rRNA gene copies of bacteria with 357F and 907R primers^111^ using the following thermal protocol: 98 °C for 3 min, followed by 40 cycles of denaturation at 98 °C for 15 s, annealing at 58 °C for 30 s, and extension at 72 °C for 1 min. Finally, a melting curve analysis was performed from 65 to 95 °C (fluorescence was measured every 0.5 °C). To evaluate whether the selected bees had a healthy status comparable to the ones shown in previous studies^42^ and to exclude dysbiotic animals from the analyses, we evaluated the organ-specific distributions of three of the main bacterial core phylotypes—*Snodgrassella*, *Lactobacillus* Firm-5, and *Gilliamella*. Their presence across the gut compartment was quantified using the primer pairs Beta-1009-qtF/Beta-1115-qtR, Firm-5-81-qtF/Firm-5-183-qtR, and Gamma1-459-qtF/Gamma1-648-qtR, respectively^42^. The following thermal protocol was used for all reactions: 98 °C for 3 min; 40 cycles of 98 °C for 15 s, annealing at 55 °C for 15 s, and extension at 72 °C for 15 s. Finally, a melting curve analysis was performed from 65 to 95 °C (fluorescence was measured every 0.5 °C). For fungi, total copies of the fungal ITS1 were determined using ITS1f and 5.8s primers^112^. The PCR conditions consisted of an initial denaturation at 98 °C for 3 min, followed by 40 cycles of denaturation at 95 °C for 15 s, annealing at 55 °C for 30 s, and extension at 72 °C for 1 min. Finally, a melting curve analysis was performed from 65 to 95 °C (fluorescence was measured every 0.5 °C). 16S rRNA gene and fungal ITS1 fragments cloned in pGEM^®^-T Easy Vector (Promega, Milan, Italy) and pCR™II-TOPO (Invitrogen, Milan, Italy) were used as standards^111^. The copy numbers of the obtained 16S rRNA gene were normalized via division to determine the mean 16S rRNA gene copy number (GCN) corresponding to the taxonomic variability present in the honeybee gut bacterial community (bacterial OTU table; Supplementary Data 1 based on rrnDB database^113^, *n* = 4.7), whereas for *Snodgrassella*, *Lactobacillus* Firm-5, and *Gilliamella*, the published genomes were used to obtain this information (16S rRNA gene copy, *n* = 4^8^). Similarly, for fungi, the values of obtained ITS1 were divided by the mean number of ITS copies present in our community (fungal OTU table; Supplementary Data 1; based on Lofgren et al.^63^, *n* = 75.5). DNA extracted from sterile water was used as an additional control in the qPCRs. No amplification was detected in negative controls for all primers pairs (results in Supplementary Table S4).

We tested the difference in the normalized number of the total bacteria and fungi and considered it as an explanatory categorical variable in the “gut compartment” (the crop, midgut, ileum, and rectum) and the “taxon” (total bacteria and fungi), which were both fixed and orthogonal. Additionally, we evaluated the differences among the three bacterial species (*Snodgrassella*, *Lactobacillus* Firm-5, and *Gilliamella*) within each gut compartment. In this case, we considered only the factor “gut compartment” as an explanatory variable. A pairwise test was performed using the R package multicomp^114^.

### Metabarcoding analysis of the bacterial 16S rRNA and fungal ITS of forager honeybee microbial community

Illumina libraries were prepared using the Illumina^®^ Nextera XT Sample Prep Kit and amplifying the V3 and V4 variable regions of the 16S rRNA gene (341F and 785R primers^115^) from both DNA and cDNA as well as the internal transcribed spacer 2 (ITS2) region (ITS3F and ITS4R primers^85^) from DNA, following the protocol described in Marasco et al.^115^. All primers used contained an adapter for the sequencing platform and an 8-nucleotide barcode. A blank control of PCR and DNA/RNA extraction reagents was performed and used to incorporate the sequencing adapters. Amplification reactions were purified and normalized using the SequalPrep™ Normalization Plate Kit (Invitrogen). All tagged samples were pooled together and concentrated in a CentriVap DNA Concentrator (Labconco). The obtained 16S rRNA and fungal ITS2 libraries were sequenced using the MiSeq system with 2 × 300 base-pair read length from the Biological Core Lab at KAUST, Saudi Arabia. For both 16S rRNA and ITS fragment datasets, sequence analyses were performed using a combination of the UPARSE v8^116^ and QIIME version 1.9^117^. Raw forward and reverse reads for each sample were assembled into paired-end reads using the fastq-join method within QIIME (minimum overlapping of 30 nucleotides and maximum 1 mismatch within the region). The paired reads were then quality filtered (no ambiguous base calls with quality values of < 20 Phred Q score). The primer sequences were removed, and the individual sample files were merged into a single fasta file. OTUs were determined at 97% similarity threshold (OTU_97_) in UPARSE. OTUs with less than two observations (singletons) were eliminated; chimeras were removed using both de novo and reference “Gold” database detection. Representative sequences of each OTU_97_ were aligned with the database in QIIME using uclust^118^ and blast commands to search against the SILVA version 138^119^ and UNITE^120^ databases for bacteria and fungi, respectively. OTU_97_ tables were created (i.e., sample ID, OTU_97_ count matrix, and relative taxonomic affiliation of each OTU_97_) for bacteria (total and active values from DNA and cDNA, respectively) and fungi. Taxonomic affiliations among the core phylotypes were confirmed creating a phylogenetic tree using the sequence available in the public database (Supplementary Table S3). OTUs present in blank controls were removed from the dataset (Supplementary Table S4). Details on the raw read processing are provided in Supplementary Table S13. Raw sequences were deposited in the Sequence Read Archive of NCBI under BioProjects PRJNA422176, PRJNA632549, and PRJNA422177.

Abundance of reads obtained from DNA sequencing were normalized. The relative abundance of each bacterial OTU along the samples was divided for the mean 16S rRNA GCN of the corresponding taxonomic level available for that OTU, which was obtained from the rrnDB database^113^ and published genomes^8^ (Supplementary Data 1). For fungi, the ITS relative abundance scores were normalized using the copy number available in literature for the Ascomycota, Basidiomycota, and Zygomycota phyla^63^ (*n* = 58, *n* = 113, and *n* = 116, respectively); for unclassified fungal phyla, the reads where normalized for the mean number of ITS detected in all analyzed fungi^63^ (*n* = 99; Supplementary Data 1). Normalized OTU tables were used for further analysis.

To test the microbial (bacteria and fungi) compositional differences along the gut compartments of the bees, we performed a permutational multivariate analysis of the variance (PERMANOVA), considering the “gut compartment” fixed and orthogonal as categorical explanatory variables. Homogeneity of the dispersions among the categorical variables was a priori tested using PERMDISP. To visualize the beta-diversity of the normalized compositional Bray–Curtis matrices of bacteria and fungi, we used principal coordinates analysis (PCoA) and CAP. The statistical tests, PCoA, and CAP were performed using PRIMER v. 6.1 and PERMANOVA+ for PRIMER routines^121^. Correlations between microbial (core bacteria or other potential environmental bacteria and fungi), beta-diversity, and physico-chemical gut conditions were determined by performing a Mantel test using Spearman’s rank correlation with the R package vegan^122^. To explore the discriminant OTUs that were affected by changes in the physico-chemical conditions along the gut compartments, the *manyglm*() function of the R package mvabund^123^ was employed using the negative binomial error distribution. We selected the best model. Each discriminant OTU was detected using a significant univariate general linear model with a negative binomial error distribution for which the *p* values were adjusted for multiple testing using a step-down resampling procedure. We selected the response variable (i.e., OTU) with *p* value of <0.005 to detect the most significant OTU change among the variables responsible for the gut physico-chemical gradient (i.e., our explanatory variable). Further, we computed the beta-diversity components using the function *beta.div.comp* of the package adespatial^124^. The rate of decay of the similarity of the community (Bray–Curtis) along the gut compartments was evaluated for both bacterial and fungal communities; a linear regression statistical analysis was subsequently performed using a GraphPad Prism (GraphPad Software, La Jolla California USA, www.graphpad.com). Alpha diversity indices were calculated using routine DIVERSE in PRIMER^121^, and an ANOVA (Tukey’s multiple comparison test) was performed to test the differences in alpha diversity indices for the “gut compartment” using the GraphPad software. Finally, to examine the functional role of other-possibly environmental bacteria and fungi during their association with the honeybee gut, taxonomy was utilized to interfere with the bacterial metabolic functions using Tax4Fun2^125^ platform and the fungal functional guild (i.e., trophic mode) using the annotation tool FUNGuild v1.09^126^.

### Microsensor measurements

The intact whole honeybee gut was dissected from adult forager bees as previously described and gently placed on the agarose surface of customized plastic chambers filled with 2% agarose and embedded in 0.5% agarose Ringer’s solution^127^. Microsensors (Unisense, Aarhus, Denmark) were used to measure the oxygen concentration, pH, and redox potential within the different gut compartments of *A. mellifera ligustica* (Italy). Overall, 11, 12, and 16 digestive tracts were dissected for the analysis of pO_2_, pH, and redox potential, respectively. Oxygen measurements were performed using oxygen microsensors with 50-µm-diameter tips (OX-50), following a calibration at pO_2_ of 0 and 21 kPa, as previously described^127^. pH microelectrodes (PH-50) with extremely sharp customized 50-µm-diameter tips were calibrated using standard solutions of pH 4.0, 7.0, and 9.0^128^. The redox microelectrodes (RD-50) with a 50-µm-diameter tip o were calibrated using saturated quinhydrone solutions in pH standard solutions of pH 4.0 and 7.0^129^. In both cases, the electrode potentials were measured against a reference electrode (REF-RM) and an open-ended Ag-AgCl electrode with a gel-stabilized electrolyte connected to a high-impedance millivolt-meter. During the entire duration of microsensor measurements, the Veho VMS-004D USB microscope was placed in front of the embedded organ. The points in which the tip of the microsensor enter and exit the gut tissue were registered, and their difference was used to estimate the diameter (mm) of each gut compartment^39,127^. The diameter was reported as the mean ± standard deviation (Supplementary Table S12). Tukey’s multiple comparison test was used to evaluate changes among gut the compartments.

### Gut metabolites analysis

We measured the sugar and SCFA concentrations within the different gut compartments of *A. mellifera ligustica* (Italy) using high-performance liquid chromatography (HPLC), following the method reported by Zheng et al.^39^. The weights of the pooled gut compartments were measured using an analytical balance. Thereafter, six compartments were homogenized in 100 μl water, centrifuged at 15,000 × *g* for 5 min, and filtered with 0.22-μm microcentrifuge tube filters (Corning) for 10 min at 10,000 × *g*. The filtrate was acidified with H_2_SO_4_ to reach a final concentration of 10 mM. Sugars were determined by performing HPLC on 10 μl of sample using two Aminex HPX-87P columns in a series (300 mm i.d. × 7.8 mm) from Bio-Rad (Segrate, Italy) maintained at 75 °C. The HPLC consisted of an Alliance 2695 pump (Waters, Milford, MA) with a model 410 differential refractometer (Waters). Chromatographic analyses were performed using MilliQ water (Millipore, Darmstadt, Germany) as an eluent at a flow-rate of 0.60 ml/min. Sugar quantification was performed by the external standard method using aqueous solutions of melezitose, sucrose, maltose, glucose, and fructose. HPLC of organic acids was conducted by injecting 10 μl of each sample into the same HPLC apparatus equipped with a Waters 2487 UV detector. An Aminex HPX-87H column (300 mm i.d. × 7.8 mm, Bio-Rad) maintained at 50 °C was used. An eluting solvent (0.60 ml/min) contained 5 mM sulfuric acid. Quantitation of organic acids in extracts was performed at 210 nm using solutions of succinic, acetic, lactic, malic, formic, propionic, and butyric acids in 5 mM sulfuric acid as external standards. All sugars and organic acids were of analytical grade (Sigma-Aldrich, St. Louis, MO, USA). Analyses were performed in triplicate, and mean values (mM) per mg of tissue were reported. Data were processed using the Millennium 32 software (Waters). The concentration of ethanol was determined using an enzymatic assay kit (Roche, R-Biopharm Italia srl, Melegnano, Italy). PERMANOVA was used to evaluate the compartmentalization of gut metabolites as continuous response variables, whereas the gut portions were used as categorical explanatory variables that are fixed and orthogonal (four levels: crop, midgut, ileum, and rectum). Moreover, difference in each metabolite concentration along the gut was tested using ANOVA and Tukey’s multiple comparison test.

### Reporting summary

Further information on research design is available in the Nature Research Reporting Summary linked to this article.

## Supplementary information

Supplementary Information

Supplementary Data 1

Supplementary Data 2

Reporting Summary

## Data Availability

All data required to evaluate the conclusions in the paper are present in the paper and/or the Additional Information; molecular data are submitted in the European Nucleotide Archive (ENA) under accession numbers LR746502-LR746517 and LR798000-LR798072 and in the Sequence Read Archive (SRA) of NCBI under BioProjects PRJNA422176, PRJNA632549, and PRJNA422177. All data are available upon request to the authors.

## Code availability

The code used for data elaboration, graph creation, and statistical analysis related to this paper are available upon request to the authors.
