# Supplementary material for: Compartmentalization of bacterial and fungal microbiomes in the gut of adult honeybees
Source: NPJ Biofilms Microbiomes. 2021 May 7;7:42. doi: 10.1038/s41522-021-00212-9 (PMC8105395; doi:10.1038/s41522-021-00212-9)
Supplement: Supplementary file 1 — Supplementary Information [file 41522_2021_212_MOESM1_ESM.pdf]

SUPPLEMENTARY METHODS

Supplementary Method S1. Preparation of gut samples for scanning electron microscopy (SEM) visualization. Post fixation was performed rinsing three times for 15 min with 0.1 M sodium cacodylate buffer (pH 7.2) and further incubating for 1 hour at room temperature in the dark with 1% osmium tetraoxide (OsO4) solution prepared with 0.1 M sodium cacodylate buffer (pH 7.2) After post-fixation, samples were rinsed with distilled water three times for 15 min. Dehydration steps of 15 min were performed with ethanol solutions series with gradient of 30%, 50%, 70%, 90% and absolute ethanol. After reaching the last step, samples were rinsed twice with absolute ethanol for 15 min. Drying of the organs was performed through evaporation of hexamethyldisilazane (HMDS) with steps of 15 min using gradually increasing concentration of HMDS in absolute ethanol (33%, 66% and 100% HMDS) and the last step was repeated for 1 h. Samples submerged in the final 100% HMDS solution were left in a fume hood at room temperature until all the HMDS solution had evaporated. Dried guts were microdissected under stereomicroscope using sterile scalpels and mounted using sterile forceps on a double-sided adhesive carbon tape placed on to aluminium stubs and coated with a 5 nm layer of Au/Pb using a K575X sputter coater (Quorum).

Supplementary Method S2. Sample preparation and visualization at transmission electron microscopy (TEM). Electron microscopy was performed on dissected gut portions from freshly honey bees collected from Makkah and Madinah regions (Saudi Arabia) after surface sterilization of the honeybee bodies. Dissected guts were fixed in a solution of 2.5% (v/v) glutaraldehyde in 0.1 M sodium cacodylate buffer (pH 7.2) and stored at 4°C. Then the samples were rinsed three times for 5 minutes with 0.1 M sodium cacodylate buffer (pH 7.2) and were post-fixed with osmium tetroxide (1%) for 1 hour at room temperature. Samples were washed three times with sterile distilled water, dehydrated in a graded series of ethanol, and incubated in propylene oxide-Epon (1:1, v/v) for 1 hour. Samples were transferred into fresh Epon in molds and cured in the oven overnight at 60°C. Ultrathin-sections were stained with uranyl acetate and examined with Titan Cs Image_iac transmission electron microscope at the KAUST Imaging and Characterization Core Lab.

Supplementary Method S3. Preparation of yeast cells for scanning electron microscopy (SEM) visualization. The yeast pellets obtained were resuspended in a sterile filtered solution of 2.5% (v/v) glutaraldehyde in 0.1 M sodium cacodylate buffer (pH 7.2) and fixed at 4°C overnight. After fixation, yeast samples were centrifuged and resuspended three times in washing steps with sterile filtered 0.1 M sodium cacodylate buffer (pH 7.2). 500 µl of the resuspended yeast cells were placed on glass coverslips pre-treated with poly-L-lysine and incubate in a six wells plate for an overnight at room temperature. Dehydration steps of 15 min were performed with ethanol solutions series with gradient of 30%, 50%, 70%, 90% and absolute ethanol. After reaching the last step, samples were rinsed twice with absolute ethanol for 15 min. Drying was then performed using a Leica EM CPD300 for the critical point drying with CO_2 and then the coverslips were glued on aluminium stubs and coated with a 5 nm layer of Au/Pb using a K575X sputter coater (Quorum).

Supplementary Method S4. RNA extraction and cDNA synthesis. After surface sterilization and sterile dissection procedures, as previously described in the Honey bees' gut dissection paragraph, we used the RNeasy Mini kit (Qiagen) on the single entire guts homogenized in 350 µl Buffer RLT using sterile plastic pestles and adding approximately 500 µl of sterile acid-washed glass beads 425-600 µm-diameter (Sigma) for a vortex step with the maximum speed for 5 minutes, then we followed the manufacture instructions. blank of extraction was also included. DNase I digestion of the extracted total RNA was performed for all the samples following the manufacture instruction of the RNeasy Mini kit (Qiagen). The concentration of the extracted total RNA was evaluated using the Qubit™ RNA broad range (BR) kit (Invitrogen) whereas the eventual contamination of gDNA was checked using the Qubit™ dsDNA high-sensitivity (HS) kit (Invitrogen).

SUPPLEMENTARY TABLES

Supplementary Table S1. (**a**) Pairwise comparison to evaluate changes of bacterial (16S rRNA gene-based) and fungal (ITS1-based) normalized-abundance (see materials and methods for details) along the gut compartments. (**b**) Pairwise comparison among total bacteria and fungi, and among the core bacterial phylotypes ($^{\#}$: *Snodgrasella*, *Lactobacillus* Firm-5 and *Gilliamella*) per each gut compartment.

(**a**) Differences of bacterial and fungal abundance along the gut compartments. * = $p < 0.05$

**Gut compartment**	**Bacteria**		**Fungi**	
	**T**	***P***	**T**	***P***
Crop, Midgut	-0.739	0.87995	-0.099	0.9996
Crop, Ileum	-5.349	< 0.001*	-2.433	0.123
Crop, Rectum	-7.76	< 0.001*	-2.942	0.0498*
Midgut, Ileum	-4.61	0.00149*	-2.536	0.1037
Midgut, Rectum	-7.021	< 0.001*	-3.071	0.0418*
Ileum, Rectum	-2.411	0.11493	-0.701	0.8943

(**b**) Differences among bacterial and fungal abundance, and among the three-core bacterial phylotypes$^{\#}$ in each gut compartment. * = $p < 0.05$

	**Bacteria vs Fungi**		**Core bacterial phylotypes$^{\#}$**	
**Gut compartment**	**t**	***P***	**F**	***P***
Crop	5.597	0.0008*	8.12	0.006*
Midgut	8.54	<0.0001*	0.17	0.85
Ileum	16.46	<0.0001*	4.48	0.037*
Rectum	13.64	<0.0001*	16.83	0.0003*

Supplementary Table S2. PERMANOVA pairwise comparison of total bacterial, other-possibly environmental bacterial, and fungal communities inhabiting the different gut portions. Bray–Curtis matrices based on OTUs tables normalized for the number of 16Sr RNA gene and ITS for bacteria and fungi, respectively (see details of normalization in materials and methods). Star (*) indicates the significant pair-comparison showing $p < 0.05$.

**Gut compartment**	**Bacteria, *p*-value**	**Other bacteria, *p*-value**	**Fungi, *p*-value**
Crop, Ileum	0.002*	0.005*	0.004*
Crop, Rectum	0.003*	0.004*	0.002*
Crop, Midgut	0.001*	0.003*	0.009*
Ileum, Rectum	0.015*	0.039*	0.1
Ileum, Midgut	0.658	0.483	0.085
Rectum, Midgut	0.003*	0.028*	0.186

Supplementary Table S3. Reference sequences used to define the OTUs belonging to the bacterial phylotypes previously found in association with the honey bee gut.

Phylotype	Species/Genus name	Acc. Num.	Reference
**Beta**	*Snodgrassella alvi*	AY370189	(Martinson *et al.*, 2012)
		NR_118404	(Kwong *et al.*, 2014)
**Gamma-1**	*Gilliamella apicola*	AY370191	(Martinson *et al.*, 2012)
		NR_121727	(Ludvigsen *et al.*, 2015)
**Firm-4**	*Lactobacillus* sp.	HM112055	(Leonhardt and Kaltenpoth, 2014)
		HM534810	(Leonhardt and Kaltenpoth, 2014)
		DQ837632	(Leonhardt and Kaltenpoth, 2014)
		HM046576	(Leonhardt and Kaltenpoth, 2014)
		EU753690	(Leonhardt and Kaltenpoth, 2014)
		HM113222	(Leonhardt and Kaltenpoth, 2014)
**Firm-5**	*Lactobacillus* sp.	GU233458	(Leonhardt and Kaltenpoth, 2014)
		AJ971929	(Leonhardt and Kaltenpoth, 2014)
		HM534786	(Leonhardt and Kaltenpoth, 2014)
		HM113203	(Leonhardt and Kaltenpoth, 2014)
		HM112866	(Leonhardt and Kaltenpoth, 2014)
		DQ837634	(Leonhardt and Kaltenpoth, 2014)
		HM046579	(Leonhardt and Kaltenpoth, 2014)
		HM113281	(Leonhardt and Kaltenpoth, 2014)
**Bifidobacterium**	*Bifidobacterium* sp.	HM108346	(Martinson *et al.*, 2011)
**Gamma-2**	*Frischella perrara*	JX878306	(Engel *et al.*, 2013)
**Alpha2.1**	*Commensalibacter* sp.	CP033087	(Siozios *et al.*, 2019)
**Alpha2.2**	*Bombella apis*	KU534110	(Yun *et al.*, 2017)
**Alpha1**	*Bartonella apis*	KP987884	(Kešnerová *et al.*, 2016)
**Apibacter**	*Apibacter*	DQ837638	(Babendreier *et al.*, 2007)

Supplementary Table S4. Negative control samples included to account for contaminations during the experimental workflow. Controls were performed starting from the blank of DNA/RNA extraction done with sterile water as sample; these samples were further used throughout all the analyses performed (quantification, amplification, and sequencing); quantification/results obtained by blanks were reported for nucleic acids extractions, qPCR, amplicon and index PCR amplification, and MiSeq sequencing reads analysis.

Analysis	
Blank DNA extraction	Under detection limit (Qubit dsDNA BR, <1 ng/ µl)
Blank RNA extraction	Under detection limit (Qubit RNA BR, <1 ng/ µl)
Blank cDNA synthesis	Under detection limit (Qubit dsDNA HS, <10 pg/µl)
Blank in qPCR bacterial 16S rRNA gene	Under detection limit (cq=36.06±0.135)
Blank in qPCR fungal ITS1	Under detection limit (cq=36.49±0.084)
PCR amplification of 16S rRNA gene from blank DNA	Band not visible in the gel 1% agarose
PCR amplification of 16S rRNA gene from blank cDNA	Band not visible in the gel 1% agarose
PCR amplification of ITS2 from blank DNA	Band not visible in the gel 1% agarose
Index PCR of bacterial amplicon from blank DNA	Band not visible in the gel 1.2% agarose
Index PCR of bacterial amplicon from blank cDNA	Band not visible in the gel 1.2% agarose
Index PCR of fungal amplicon from blank DNA	Band not visible in the gel 1.2% agarose
Bacterial OTUs in blank DNA	7 OTUs, total 333 reads removed from the dataset
Bacterial OTUs in blank cDNA	4 OTUs, total 7 reads removed from the dataset
Fungal OTUs in blank DNA	6 OTUs, total 1691 reads removed from dataset

Supplementary Table S5. Taxonomy and distribution of bacterial and fungal OTUs along the gut compartments of nurse honeybees from Saudi Arabia is resumed. For bacteria categories of core bacteria and other-possibly environmental bacteria are listed; distribution and abundance of fungi are reported at genus level. Normalized reads are expressed as percentage of average relative abundance (n = 5).

**Bacterial category**	**N. OTU**	**Av. relative abundance (%)**			
		Crop	Midgut	Ileum	Rectum
Snodgrassella	1	0.41	0.68	7.12	0.35
Gilliamella	3	5.60	19.79	42.45	6.79
Lactobacillus Firm-4	3	1.50	1.06	1.76	14.82
Lactobacillus Firm-5	11	15.04	30.07	19.08	43.74
Bifidobacterium	1	0.10	0.11	6.96	25.61
Bartonella	1	1.16	1.37	15.88	4.13
Frischella	1	0.37	4.89	6.19	0.76
Apibacter	1	0	0	0.002	0.004
Bombella	4	1.47	0.13	0.001	0.00
Commensalibacter	1	0.004	0	0.001	0.07
Other *Enterobacteriaceae*	48	21.60	12.05	0.05	0.03
Other *Lactobacillus* sp.	81	3.07	1.75	0.15	3.33
Others	114	49.68	28.10	0.37	0.36

**Fungi (genera)**	**N. OTUs**	**Av. relative abundance (%)**			
		**Crop**	**Midgut**	**Ileum**	**Rectum**
Alternaria	5	19.47	17.07	2.74	19.73
Aspergillus	12	9.90	14.68	18.32	9.56
Aureobasidium	2	0.45	0.00	7.26	0.97
Cladosporium	2	11.31	6.21	4.32	7.11
Eremothecium	2	10.87	5.82	19.77	11.65
Fusarium	6	7.98	5.47	2.76	6.19
Leucoagaricus	1	1.06	0.02	12.89	1.69
Macrophomina	1	1.98	2.57	0.07	2.56
Tulostoma	2	0.50	0.04	19.51	0.27
Zygosaccharomyces	1	23.72	36.73	9.29	23.84
Other Fungi (43 genera < 1% and ucl.)	92	12.78	11.40	3.08	16.43

Supplementary Table S6. Sample location of the honey bees (*Apis* spp.) collected at the hive used in this work. Number of honey bees' specimens used for each experiment are reported. Star (*) indicate when organs were pooled during the analysis; # bacterial 16S rRNA gene amplification.

Group	Species/Subspecies	Country	City/Region	Latitude	Longitude	Date (MM/YY)
A	*A. mellifera ligustica*	IT	Grugliasco, Piedmont	45.066874	7.590442	09/2016
B	*A. mellifera ligustica*	IT	Grugliasco, Piedmont	45.066874	7.590442	10/2018
C	*A. mellifera jemenitica*	KSA	Jeddah, Makkah	21.484389	39.194639	01/2020
D	*A. mellifera jemenitica*	KSA	Bryman, Makkah	21.647138	39.248959	01/2020
E	*A. mellifera jemenitica*	KSA	Al Musayjid, Madinah	24.087309	39.095556	02/2020
F	*A. florea*	KSA	KAUST (Thuwal), Makkah	22.326796	39.109266	02/2020

Group	MiSeq gDNA	MiSeq cDNA#	qPCR	Microsensors profiling	Metabolites analysis	Fungal isolation	Microscopy (SEM)
A	50*	-	50*	40	18*	-	-
B	-	-	-	-	-	9*	-
C	-	-	-	-	-	3	-
D	-	-	-	-	-	12*	3
E	50*	6	-	-	-	9*	3
F	-	-	-	-	-	9*	-

Supplementary Table S7. Phylogenetic identification of 90 culturable fungi randomly selected from the colonies isolated from the *Apis*. spp. gut (Supplementary Table 8). Species of *Apis* used, code of the isolate, closest described species for the ITS1 sequence obtained from NCBI with relative accession number and percentage of identity are reported for each fungal isolate. Star (*) indicate the two strains selected for the visualization at the scanning electron microscope (SEM, see Figs. 1g, h and Supplementary Fig. S9).

**Source**	**Isolate code**	**Closest described species**	**Acc. Num.**	**Length (bp)**	**% id.**
A. mellifera ligustica	L3	*Starmerella bombicola*	HQ111055	143/144	99
A. mellifera ligustica	L4	*Starmerella bombicola*	HQ111055	136/136	100
A. mellifera ligustica	L9	*Starmerella bombicola*	HQ111055	143/144	99
A. mellifera ligustica	L16	*Hanseniaspora uvarum*	MN587889	322/324	99
A. mellifera ligustica	L17	*Hanseniaspora uvarum*	MN587889	323/325	99
A. mellifera ligustica	**L18***	***Hanseniaspora uvarum***	**MN587889**	**322/324**	**99**
A. mellifera ligustica	L24	*Hanseniaspora uvarum*	MN587884	322/324	99
A. mellifera ligustica	L25	*Hanseniaspora uvarum*	MN587889	324/326	99
A. mellifera ligustica	L26	*Hanseniaspora uvarum*	MN587889	323/325	99
A. mellifera ligustica	**L28***	***Starmerella bombicola***	**HQ111044**	**137/137**	**100**
A. mellifera ligustica	L29	*Starmerella bombicola*	KY105543	136/136	100
A. mellifera ligustica	L30	*Starmerella bombicola*	KY105543	134/134	100
A. mellifera ligustica	L35	*Starmerella bombicola*	KY105543	142/143	99
A. mellifera ligustica	L36	*Starmerella bombicola*	HQ111044	144/145	99
A. mellifera ligustica	L39	*Hanseniaspora uvarum*	MN587889	323/325	99
A. mellifera ligustica	L40	*Hanseniaspora uvarum*	MN587884	322/324	99
A. mellifera jemenitica	JSU3-1	*Starmerella meliponinorum*	AB568365.1	160/162	99
A. mellifera jemenitica	JSU3-2	*Naganishia diffluens*	MN826141.1	193/193	100
A. mellifera jemenitica	JSU3-3	*Naganishia diffluens*	MN826141.1	185/185	100
A. mellifera jemenitica	JSU3-4	*Naganishia diffluens*	MN826141.1	186/186	100
A. mellifera jemenitica	JSU3-5	*Naganishia diffluens*	MN826141.1	176/176	100
A. mellifera jemenitica	JSU3-6	*Aureobasidium pullulans*	MT108187.1	198/198	100
A. mellifera jemenitica	JSU3-7	*Aureobasidium pullulans*	MT108187.1	209/209	100
A. mellifera jemenitica	JSU3-8	*Naganishia diffluens*	MN826141.1	185/185	100
A. mellifera jemenitica	JSU3-9	*Naganishia diffluens*	MN826141.1	185/185	100
A. mellifera jemenitica	JSU3-10	*Aureobasidium pullulans*	MT108187.1	208/208	100
A. mellifera jemenitica	JSU3-11	*Naganishia diffluens*	MN826141.1	161/161	100
A. mellifera jemenitica	JSU3-12	*Naganishia diffluens*	MN826141.1	176/176	100
A. mellifera jemenitica	JSU3-13	*Aureobasidium pullulans*	MT108187.1	199/199	100
A. mellifera jemenitica	JSU3-14	*Naganishia diffluens*	MN826141.1	183/183	100
A. mellifera jemenitica	JSU3-15	*Hanseniaspora guilliermondii*	MG871197.1	193/193	100
A. mellifera jemenitica	JSU3-52	*Aureobasidium pullulans*	MT108187.1	134/134	100
A. mellifera jemenitica	JSU3-54	*Naganishia diffluens*	MN826141.1	185/185	100
A. mellifera jemenitica	JSL3-24	*Chaetomium brasiliense*	KX146504.1	196/196	100
A. mellifera jemenitica	MSL1-1	*Chrysosporium sp.*	MK361133.1	249/250	99
A. mellifera jemenitica	MSL3-30	*Gymnoascus dankaliensis*	MH860564.1	236/236	100
A. mellifera jemenitica	MSL3-32	*Gymnoascus dankaliensis*	MH860564.1	234/234	100
A. mellifera jemenitica	MSL3-39	*Gymnoascus dankaliensis*	MH860564.1	236/236	100
A. mellifera jemenitica	MSL3-40	*Starmerella meliponinorum*	AB568365.1	160/162	99
A. mellifera jemenitica	MSL3-41	*Starmerella meliponinorum*	AB568365.1	159/159	100
A. mellifera jemenitica	MED-A1-2	*Moniliella pollinis*	MK027073.1	109/109	100
A. mellifera jemenitica	MED-A1-3	*Aspergillus niger*	MT103092.1	171/171	100
A. mellifera jemenitica	MED-A1-4	*Penicillium citrinum*	MN535093.1	116/116	100
A. mellifera jemenitica	MED-A1-5	*Rhizopus microsporus*	KY260682.1	220/220	100
A. mellifera jemenitica	MED-A1-7	*Aureobasidium pullulans*	MT108187.1	148/148	100

A. mellifera jemenitica	MED-A1-9	*Penicillium georgiense*	KX507077.1	150/150	100
A. mellifera jemenitica	MED-A1-10	*Aspergillus niger*	MT103092.1	177/177	100
A. mellifera jemenitica	MED-A2-14	*Aureobasidium pullulans*	MT108187.1	140/140	100
A. mellifera jemenitica	MED-A3-11	*Aureobasidium pullulans*	MT108187.1	133/133	100
A. mellifera jemenitica	MED-A3-12	*Moniliella pollinis*	MK027073.1	106/106	100
A. mellifera jemenitica	MED-P1-15	*Moniliella pollinis*	MK027073.1	103/103	100
A. mellifera jemenitica	MED-P1-17	*Moniliella pollinis*	MK027073.1	109/109	100
A. mellifera jemenitica	MED-P1-19	*Aspergillus niger*	MT103092.1	190/190	100
A. mellifera jemenitica	MED-P1-21	*Moniliella pollinis*	MK027073.1	109/109	100
A. mellifera jemenitica	MED-P2-22	*Aspergillus niger*	FJ537110.1	179/179	100
A. mellifera jemenitica	MED-P2-23	*Aspergillus niger*	MG228419.1	168/168	100
A. mellifera jemenitica	MED-P3-24	*Penicillium chrysogenum*	MN518390.1	174/174	100
A. florea	AFKG-1	*Starmerella meliponinorum*	AB568365.1	125/125	100
A. florea	AFKG-2	*Starmerella meliponinorum*	AB568365.1	164/164	100
A. florea	AFKG-3	*Starmerella meliponinorum*	AB568365.1	109/109	100
A. florea	AFKG-4	*Starmerella meliponinorum*	AB568365.1	120/120	100
A. florea	AFKG-5	*Starmerella meliponinorum*	AB568365.1	117/117	100
A. florea	AFKG-7	*Starmerella meliponinorum*	AB568365.1	108/108	100
A. florea	AFKG-8	*Chaetomium sp.*	MT089953.1	145/145	100
A. florea	AFKG-9	*Aspergillus unguis*	MT003976.1	114/114	100
A. florea	AFKG-11	*Aspergillus unguis*	MT003976.1	111/111	100
A. florea	AFKG-13	*Aspergillus niger*	MG228419.1	137/137	100
A. florea	AFKG-15	*Aspergillus niger*	MT103092.1	149/149	100
A. florea	AFKG-16	*Aspergillus unguis*	MT003976.1	119/119	100
A. florea	AFKG-17	*Penicillium citrinum*	MN535093.1	114/114	100
A. florea	AFKG-18	*Cephalotheca foveolata*	KT385713.1	105/105	100
A. florea	AFKG-19	*Cephalotheca foveolata*	KT385713.1	137/137	100
A. florea	AFKG-20	*Starmerella meliponinorum*	AB568365.1	164/164	100
A. florea	AFKG-21	*Starmerella meliponinorum*	AB568365.1	117/117	100
A. florea	AFKG-22	*Starmerella meliponinorum*	AB568365.1	111/111	100
A. florea	AFKG-23	*Starmerella meliponinorum*	AB568365.1	117/117	100
A. florea	AFKG-24	*Starmerella meliponinorum*	AB568365.1	100/100	100
A. florea	AFKG-105	*Starmerella meliponinorum*	AB568365.1	148/148	100
A. florea	AFKG-114B	*Starmerella meliponinorum*	AB568365.1	160/162	99
A. florea	AFKG-115	*Starmerella meliponinorum*	AB568365.1	125/125	100
A. florea	AFKG-116	*Hanseniaspora guilliermondii*	MG871197.1	267/267	100
A. florea	AFKG-117	*Candida sp.*	HM044861.1	146/146	100
A. florea	AFKG-118	*Hanseniaspora guilliermondii*	MG871197.1	193/193	100
A. florea	AFKG-120A	*Starmerella meliponinorum*	AB568365.1	159/159	100
A. florea	AFKG-120B	*Starmerella meliponinorum*	AB568365.1	159/159	100
A. florea	AFKG-175	*Quambalaria cyanescens*	MN162007.1	188/188	100
A. florea	AFKG-176	*Starmerella meliponinorum*	AB568365.1	159/159	100
A. florea	AFKG-179	*Candida sp.*	MN832575.1	137/137	100
A. florea	AFKG-193	*Candida sp.*	MN832575.1	119/119	100
A. florea	AFKG-194	*Candida sp.*	MN832575.1	119/119	100

Supplementary Table S8. Comparison between molecular and physio-chemical data measured in Zheng *et al*. (Zheng *et al.*, 2017) and this study. Data are expressed as average and the number of replicates is specified in the table; n/a: not available data; -: not detectable.

**Molecule/parameter**	**Crop**		**Midgut**		**Ileum**		**Rectum**	
	**This study**	**Zheng *et al.***	**This study**	**Zheng *et al.***	**This study**	**Zheng *et al.***	**This study**	**Zheng *et al.***
Glucose (mM)	24	n/a	13.05	156.3	6.34	67.4	2.5	7.3
Fructose (mM)	56	n/a	12.66	81.9	0.52	5.4	1.3	4.1
Sucrose (mM)	12.22	n/a	1.22	-	0.09	-	-	-
Melezitose (mM)	4.04	n/a	0.53	n/a	-	n/a	-	n/a
Maltose (mM)	3.23	n/a	0.47	n/a	0.02	n/a	-	n/a
Maltotriose (mM)	-	n/a	-	n/a	-	n/a	-	n/a
Replicates for sugars	3	12	3	12	3	12	3	12
Acetate (mM)	-	n/a	30.53	-	11.97	114.0	38.17	136.5
Lactate (mM)	-	n/a	-	-	-	13.2	-	14.4
Succinate (mM)	7.72	n/a	18.40	-	2.14	-	5.48	36.7
Propionate (mM)	-	n/a	-	-	-	-	-	9.9
Malate (mM)	-	n/a	-	-	-	-	-	-
Formate (mM)	-	n/a	-	-	-	-	-	-
Butyrate (mM)	-	n/a	-	-	-	-	-	-
Replicates for SCFAs	3	12	3	12	3	12	3	12
pH (unit)	6.1	5.8	5.6	6.3	5.3	5.2	4.8	5.2
Oxygen (%)	0	0	0	0	0	0	0-2.8	0
Redox (mV)	292	120	266	110	296	50	293	110
Replicates for physiology	11-18	16	11-18	16	11-18	16	11-18	16

Supplementary Table S9. PERMANOVA pairwise comparison of (**a**) physico-chemical and (**b**) metabolic conditions of the four gut portions based on the related Euclidean distance matrices. Star (*) indicates the significant pair-comparison showing $p < 0.05$.

(**a**) Physico-chemical conditions

**Gut compartment**	**T**	***P***
Crop, Midgut	2.9738	0.004*
Crop, Ileum	0.6638	0.065
Crop, Rectum	3.6186	0.002*
Midgut, Ileum	3.1534	0.001*
Midgut, Rectum	3.4472	0.001*
Ileum, Rectum	3.5114	0.001*

(**b**) Metabolic (sugars and SFACs) conditions

**Gut compartment**	**T**	***P***
Crop, Midgut	3.5241	0.006*
Crop, Ileum	4.6117	0.003*
Crop, Rectum	4.6129	0.003*
Midgut, Ileum	1.776	0.001*
Midgut, Rectum	1.5411	0.091
Ileum, Rectum	2.4218	0.046*

Supplementary Table S10. Step wise model selection to determine the significant explanatory variables determining the change in (a) core bacteria, (b) other-possibly environmental bacteria and (c) fungal community, using a multivariate generalised linear model. In bold the parameter most significantly explaining the changes of community composition. No interaction among the explanatory variables were found significant.

(**a**) Core bacteria

Explanatory variable	Step	Model	Residual d.f.	Deviance	AIC	p
**Physical conditions**	0	**pH**	**18**	**5434.3**	**157.02**	**0.042**
	1	Oxygen	17	1389.8	157.09	0.683
	2	Redox	16	1746.3	159.17	0.49

Explanatory variable	Step	Model	Residual d.f.	Deviance	AIC	p
**Sugars**	0	**Sucrose**	**18**	**9566.8**	**152.61**	**0.001**
	1	Meletzsiose	17	3762.1	152.95	0.064
	2	Maltose	16	2428.8	154.35	0.199
	3	Glucose	15	2033.8	152.77	0.065
	4	Fructose	14	1230.7	155.7	0.458

Explanatory variables	p
**SFCAs**	> 0.05

(**b**) Other-possibly environmental bacteria

Explanatory variable	Step	Model	Residual d.f.	Deviance	AIC	p
**Physical conditions**	**0**	**pH**	**18**	**7039.1**	**162.01**	**0.047**
	1	Oxygen	17	1835.7	162.78	0.82
	2	Redox	16	3144.7	164.57	0.346

Explanatory variable	Step	Model	Residual d.f.	Deviance	AIC	p
**Sugars**	**0**	**Sucrose**	**18**	**3314.9**	**158.93**	**0.002**
	1	Meletzsiose	17	1131.9	160.18	0.25
	2	Maltose	16	1042	162.84	0.843
	3	Glucose	15	1315.9	164.19	0.116
	4	Fructose	14	1875.3	164.8	0.3

Explanatory variable	p
**SCFAs**	> 0.05

(**c**) Fungi

**Explanatory variable**	**p**
**Physical conditions**	> 0.05

**Explanatory variable**	**Step**	**Model**	**Residual d.f.**	**Deviance**	**AIC**	**p**
**Sugars**	**0**	**Sucrose**	**18**	**8384.7**	**162.4**	**0.009**
	1	Meletzsiose	17	3580.6	162.64	0.42
	2	Maltose	16	3100.5	164.17	0.386
	3	Glucose	15	3329.5	166.18	0.295
	4	Fructose	14	2302.8	169.17	0.506

**Explanatory variable**	**Step**	**Model**	**Residual d.f.**	**Deviance**	**AIC**	**p**
**SCFAs**	**0**	**Acetic Acid**	**18**	**5434.3**	**7830.9**	**0.027**
	1	Succinic Acid	17	1389.8	5419.3	0.078

Supplementary Table S11. SCFAs and sugars quantification expressed in mM/mg of tissues for single organ (mean ± standard error; n=3). Among SCFAs, lactate, propionate, malate, formate and butyrate were under the detection limit (0.01 mg/ml) in all gut compartments, as well maltotriose among sugar. Star (*) indicates statistically significant difference (ANOVA, p<0.05). Lowercase letters indicate results of Tukey's multiple comparison tests; n.d.: under the detection limit (0.01 mg/ml).

**Metabolite (mM)**		**Gut compartment**			
		**Crop**	**Midgut**	**Ileum**	**Rectum**
SCFAs	Acetate*	n.d.	2.1 ± 0.68 a	5.31 ± 1.04 b	2.38 ± 0.22 a
	Succinate	1.65 ± 1.35	1.2 ± 0.65	0.8 ± 0.2	0.5 ± 0.4
Sugars	Melezitose*	1.11 ± 0.46 a	0.03 ± 0.03 b	n.d.	n.d.
	Sucrose*	3.55 ± 1.45 a	0.07 ± 0.07 b	0.05 ± 0.02 b	n.d.
	Maltose	0.84 ± 0.54	0.03 ± 0.03	0.01 ± 0.01	n.d.
	Glucose*	4.66 ± 1.19 a	0.83 ± 0.39 b	0.38 ± 0.09 b	0.19 ± 0.08 b
	Fructose*	13.32 ± 4.80a	0.81 ± 0.49b	0.18 ± 0.05b	0.09 ± 0.06b

Supplementary Table S12. Weight of the honeybee gut compartments from Italian and Saudi Arabian forager bees were reported. Fresh weight is measured considering pool of 10 gut compartments; values are expressed in grams and reported as mean ± standard deviation (n = 5 pools). Organ diameter of Italian bees is measured considering one organ at time (n=12) and it is reported in millimetre as mean ± standard deviation (n=12); n.m., not measured.

**Origin and *Apis* species**	**Compartment**	**Weight (g)**	**Diameter (mm)**
Italy	Crop	0.0334 ± 0.0072	0.525 ± 0.194
A. mellifera ligustica	Midgut	0.1559 ± 0.0290	1.186 ± 0.230
	Ileum	0.0111 ± 0.0041	0.488 ± 0.138
	Rectum	0.3684 ± 0.0226	1.464 ± 0.264
Saudi Arabia	Crop	0.0068 ± 0.0038	n.m.
A. mellifera jemenitica	Midgut	0.0189 ± 0.0060	n.m.
	Ileum	0.0031 ± 0.0014	n.m.
	Rectum	0.0323 ± 0.0115	n.m.

Supplementary Table S13. Sequence analysis and OTUs selection for bacteria and fungi. For each sample, number of reads for the different categories are reported, along with the number of OTUs corresponding to that category. Good's coverage values are also listed.

Sample ID	Reads after quality check	Unassigned (23 OTUs)	Plastid (4 OTUs)	Used (196 OTUs)	Good's coverage
**C1**	97266	12	172	74827	0.9998
**C2**	87621	5	1	75719	0.9998
**C3**	76879	6	0	61473	0.9997
**C4**	88459	28	18	63353	0.9997
**C5**	101659	12	12	83720	0.9998
**M1**	68532	26	2	48272	0.9997
**M2**	96414	93	0	64659	0.9998
**M3**	90815	21	2	75344	0.9997
**M4**	80984	5	11	76715	0.9998
**M5**	18248	3	2	17956	0.9994
**I1**	116503	7	6	110070	0.9999
**I2**	26617	9	2	19382	0.9993
**I3**	76935	38	3	56157	0.9998
**I4**	70534	23	4	56142	0.9998
**I5**	71936	32	5	55202	0.9997
**R1**	91495	137	6	76058	0.9999
**R2**	99885	56	15	81331	0.9999
**R3**	69903	87	1	54390	0.9997
**R4**	73164	36	2	60731	0.9996
**R5**	29271	36	16	27075	0.9997

Sample ID	Reads after quality check	Unassigned 107 OTUs	Non-fungi* 21 OTUs	Rel. ab. <0.001% 44 OTUs	Used 118 OTUs	Good's coverage
**C1**	14813	3083	184	1	11545	0.999
**C2**	57085	6045	561	4	50475	1.000
**C3**	1658	574	187	2	895	0.991
**C4**	11966	964	150	10	10842	1.000
**C5**	20552	5975	241	4	14332	0.999
**I1**	26162	250	441	3	25468	1.000
**I2**	53196	10947	11073	12	31164	1.000
**I3**	21536	16155	1093	0	4288	0.999
**I4**	48542	394	146	4	47998	1.000
**I5**	37109	240	188	2	36679	1.000
**M1#**	110210	109787	220	3	200	0.921
**M2**	43909	5227	37857	6	819	0.990
**M3**	34985	3885	29554	2	1544	0.988
**M4**	52331	24855	17718	0	9758	0.999
**M5**	23226	2575	332	1	20318	0.999
**R1**	34388	5691	498	11	28188	0.999
**R2**	56557	7426	5351	16	43764	1.000
**R3**	51900	210	295	2	51393	1.000
**R4**	44542	831	448	5	43258	1.000
**R5**	17783	395	55	2	17331	0.999

SUPPLEMENTARY FIGURES

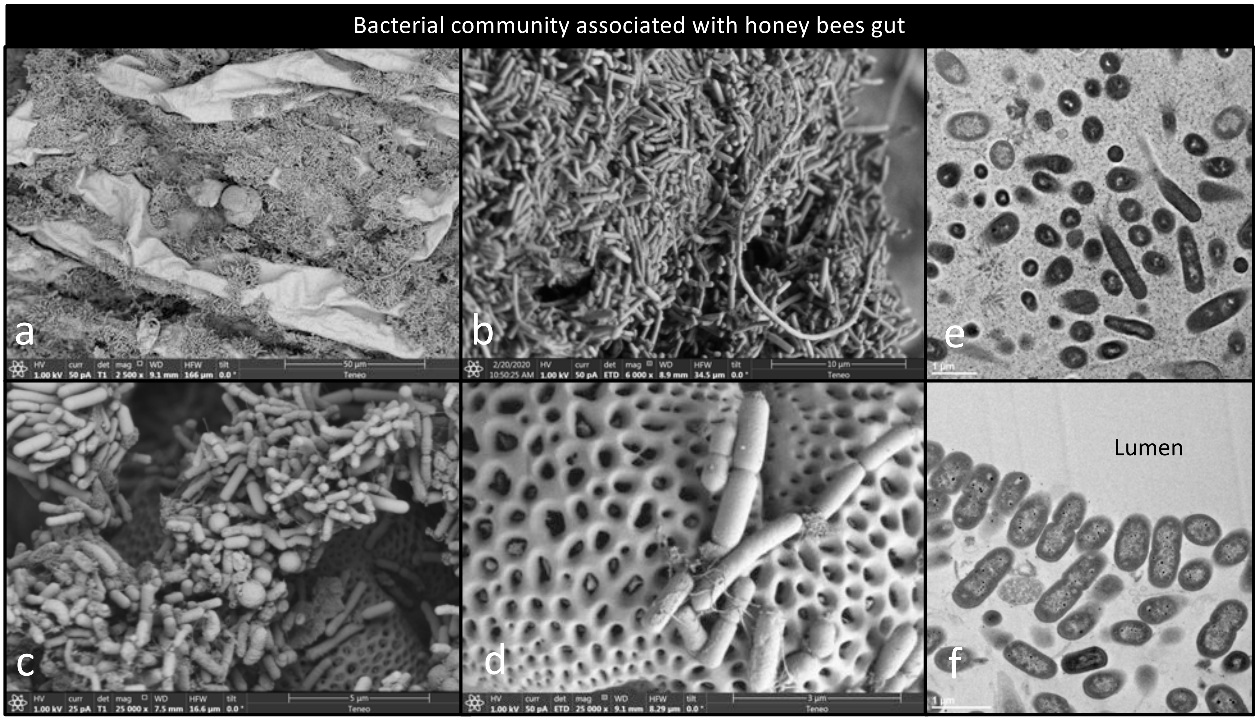

Supplementary Figure S1. (**a-d**) Bacterial members of the microbial community associated with the honey bees' gut. Honey bees used for this analysis were collected from Saudi Arabian hives. (**c** and **d**) Bacteria covering/associated with pollen grains. (**e** and **f**) Micrographs of the midgut and ileum sections, respectively, obtained using the transmission electron microscope. Bacterial cell associated with the gut epithelium are visible.

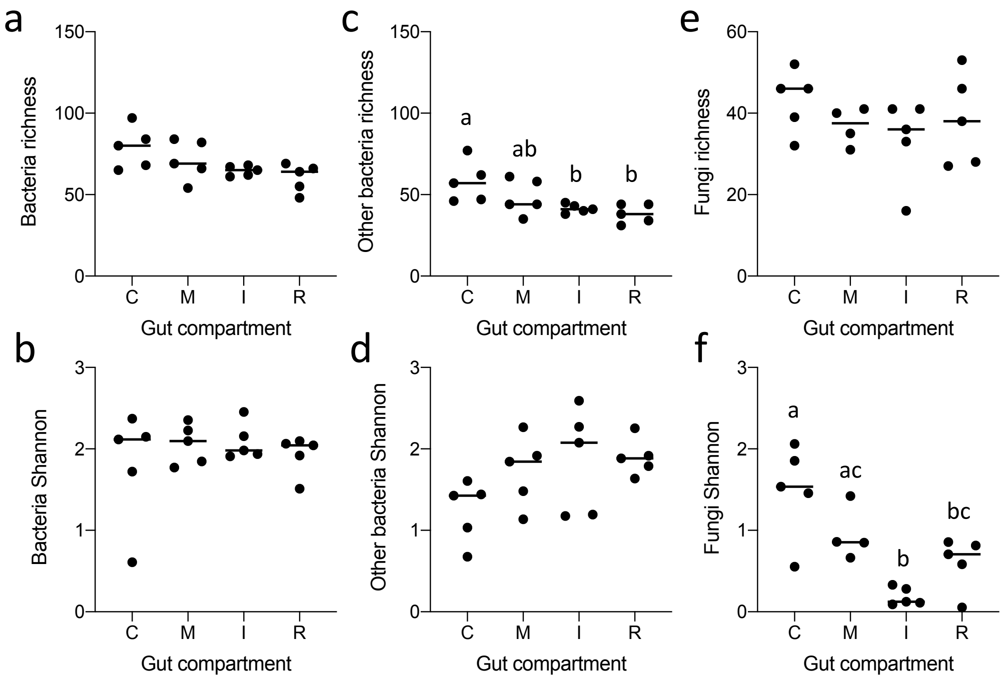

Supplementary Figure S2. Richness (number of OTUs; upper panels) and diversity (expressed as Shannon index; lower panels) for (**a** and **b**) total bacterial, (**c** and **d**) other-possibly environmental bacterial (Kwong *et al.*, 2017), and (**e** and **f**) fungal communities. Significant differences (p < 0.05) of pairwise comparison test (Tukey test) among gut compartments are indicated with lowercase letters. C, crop; M, midgut; I, ileum; R, rectum.

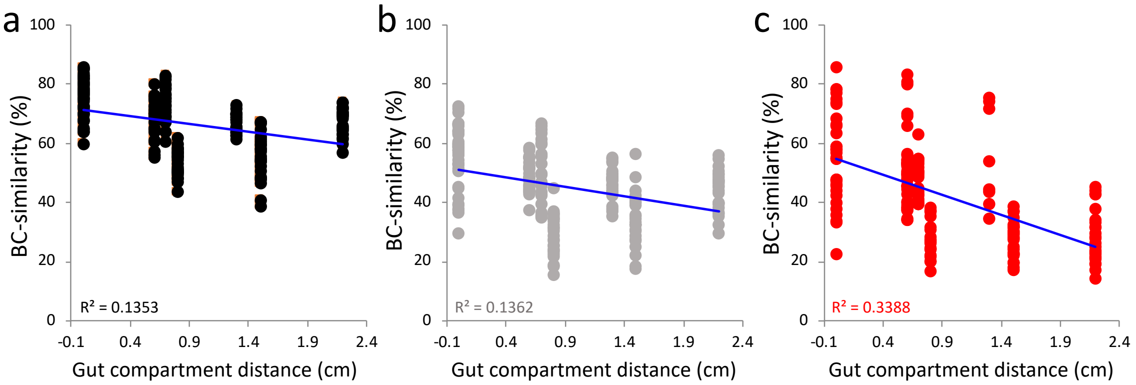

Supplementary Figure S3. Distance decay analysis of Bray-Curtis similarity across the relative distances of the gut compartments for (**a**) total bacterial, (**b**) other-possibly environmental bacterial (Kwong *et al.*, 2017), and (**c**) fungal communities, respectively. All correlations are statistically significant (Spearman, p < 0.0001).

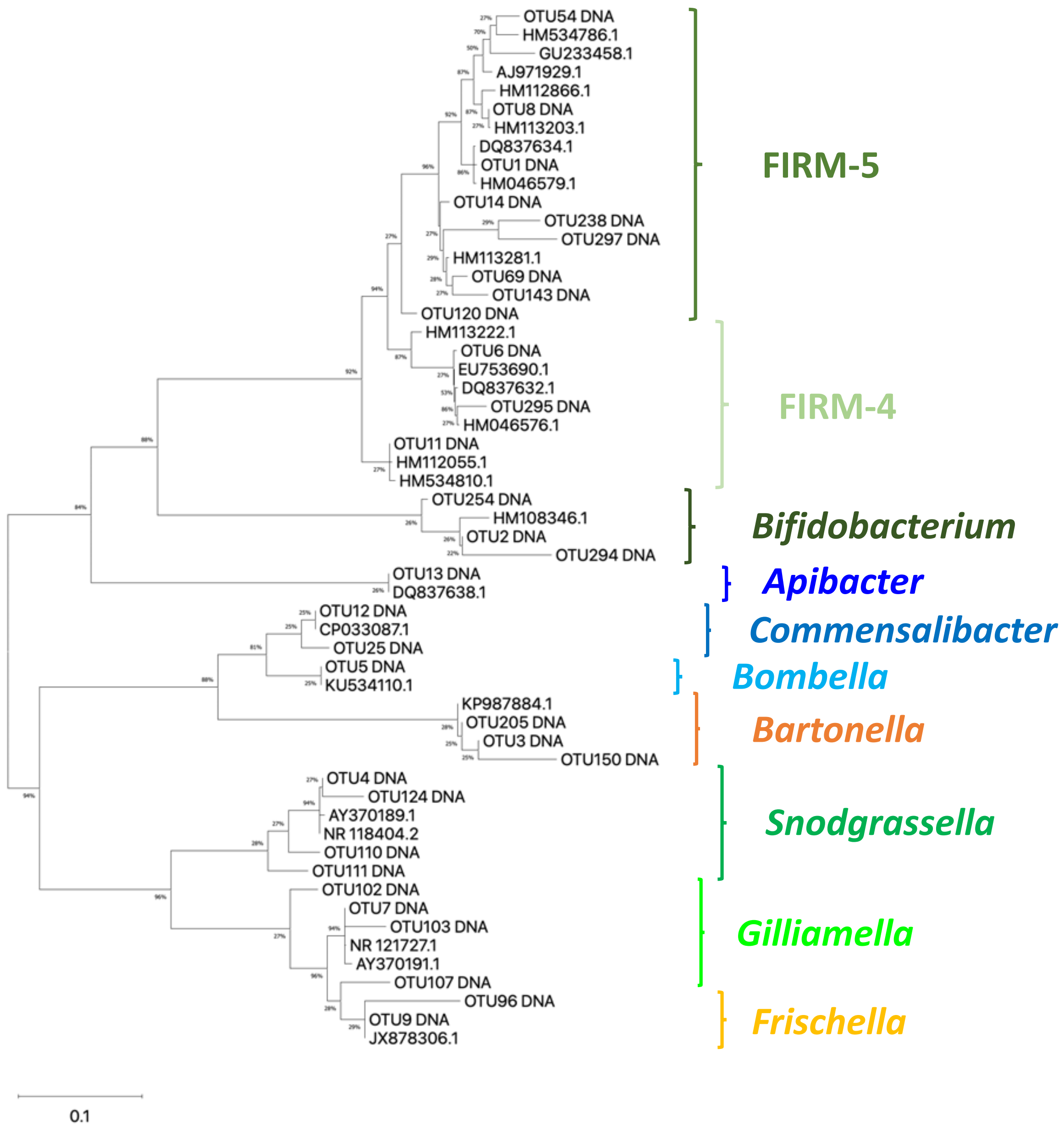

Supplementary Figure S4. Phylogenetic trees showing the 32 OTUs belonging to the 10 core bacterial taxa, obtained from DNA sequencing analysis and the reference sequences (Supplementary Table S4). OTUs of 97% sequence similarity were considered as belonging to the same phylotype (Zheng *et al.*, 2019). OTUs not included in the phylogenetic tree belong to the other-possibly environmental group of bacteria (Kwong *et al.*, 2017). The evolutionary history was inferred by using the Maximum Likelihood method and Jukes-Cantor model (Jukes and Cantor, 1969). Evolutionary analyses were conducted in MEGAX (Kumar *et al.*, 2016). Bootstrap: 1000 replicates.

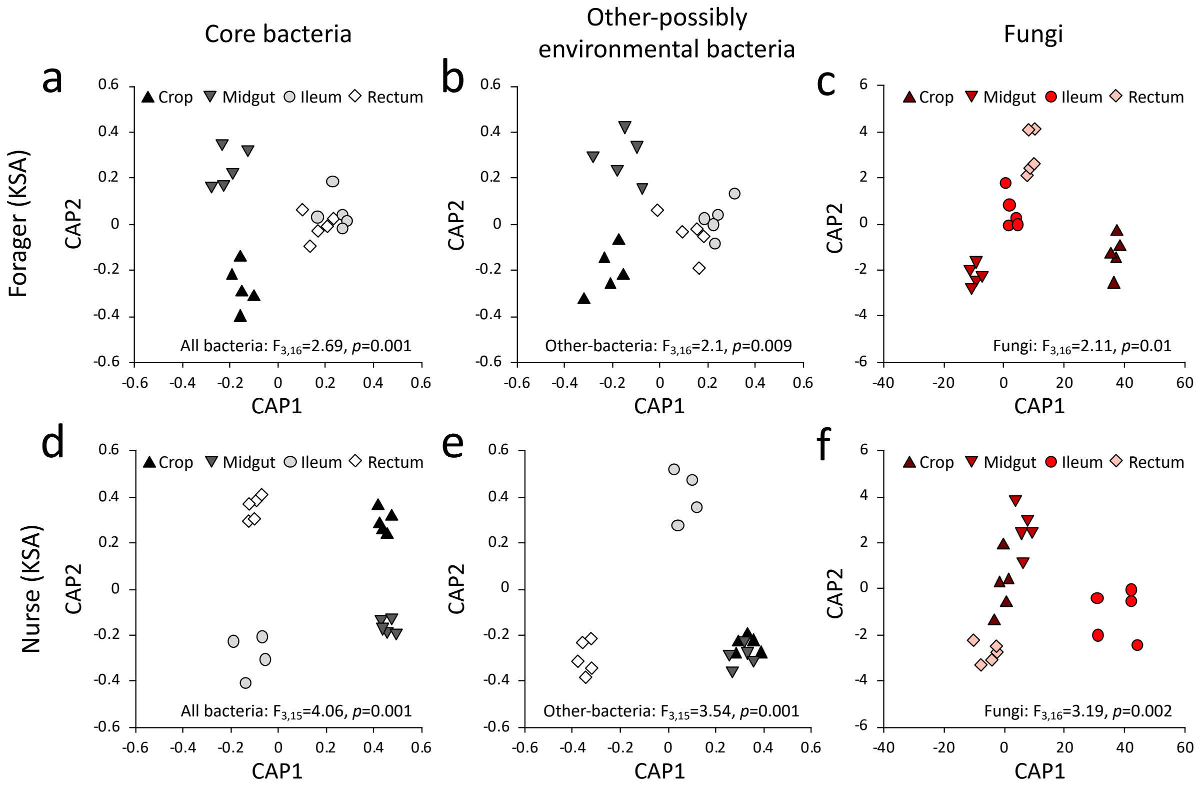

Supplementary Figure S5. Microbial communities associated with the gut compartments of *A. mellifera jemenitica* (**a-c**) forager and (**d-f**) nurse bees collected in Saudi Arabia (Madinah); beta-diversity analysis of (**a,d**) core bacterial, (**b,e**) other-possibly environmental bacterial and (**c,f**) fungal communities across the gut compartments was visualized in the space of the canonical analysis of principal coordinates (CAP). Note, data are referred to five gut compartments' pools (each pool, n = 10 individuals).

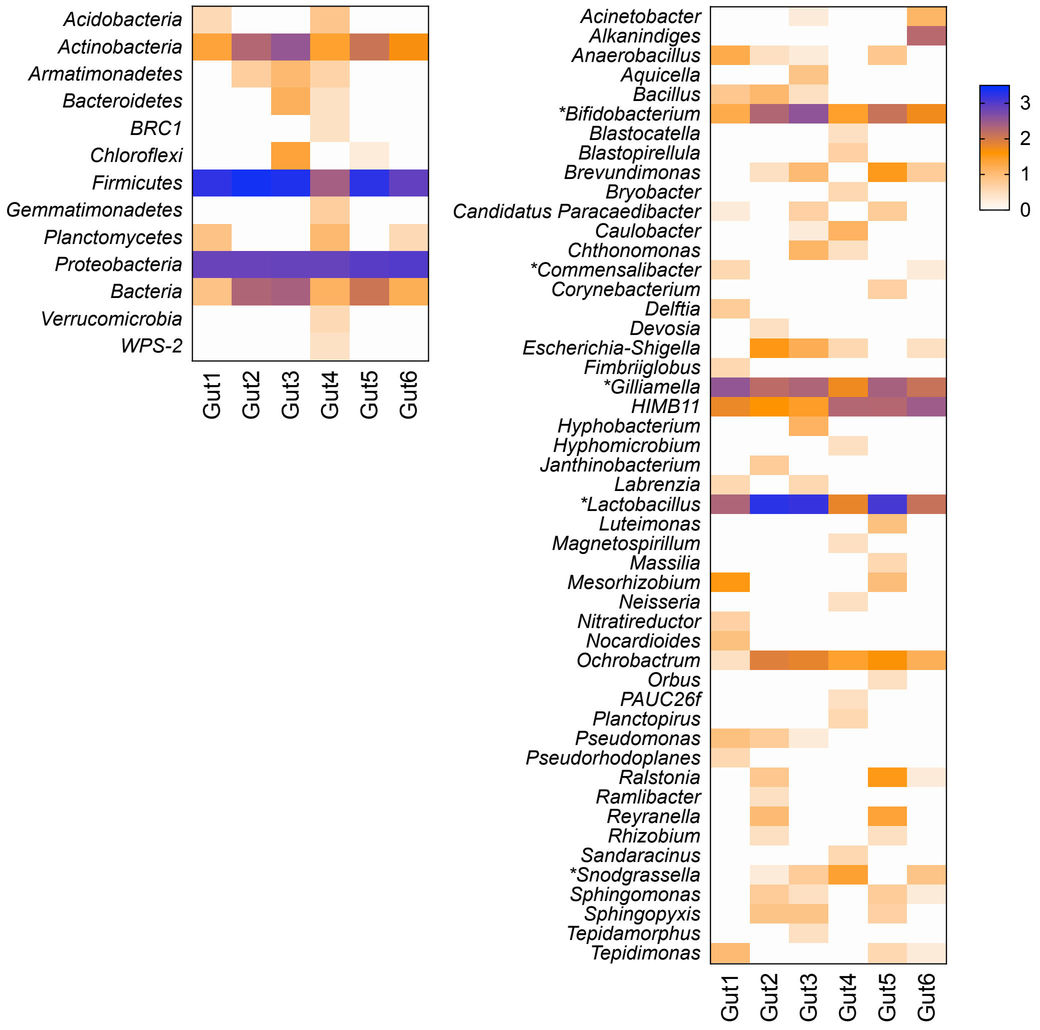

Supplementary Figure S6. Heat map represent the distribution of the other-possibly environmental bacteria metabolically active in the honey bee gut; values are reported relative abundance at phylum and genus levels. Star (*) indicate OTUs affiliate to genus typical of core-microbiome but not recognized within that clades (< 97% identity).

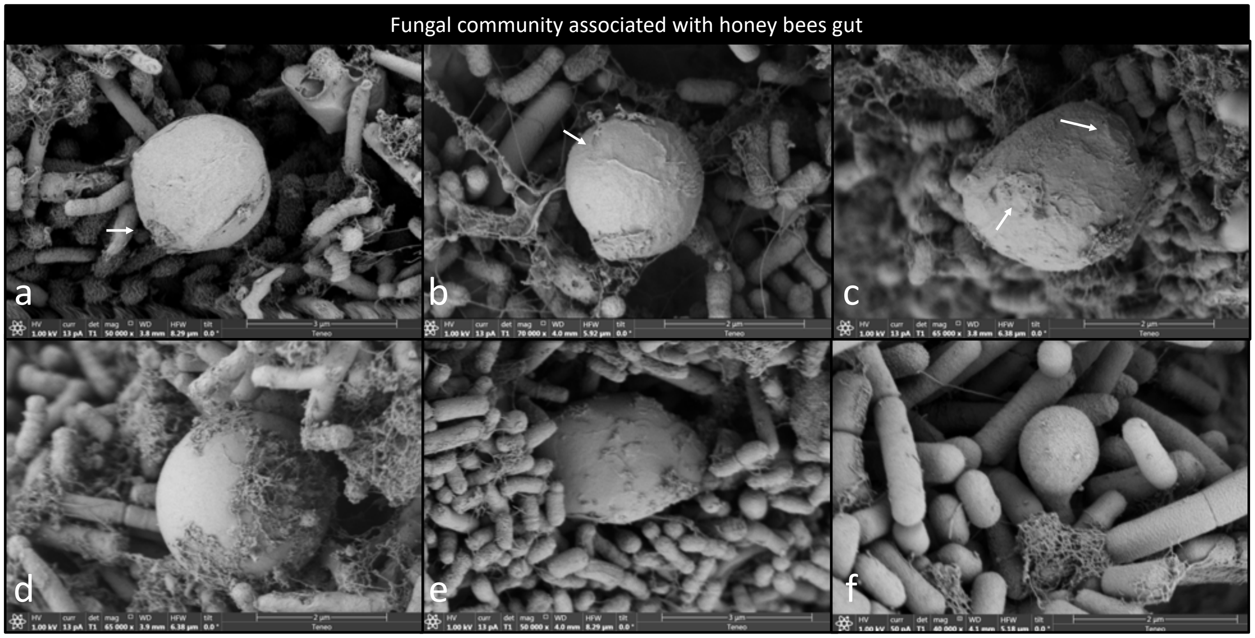

Supplementary Figure S7. Members of fungal communities (mainly yeasts) associated with the honey bees' gut. Honey bees used for this analysis were collected from Saudi Arabian hives. (**a, b** and **c)** Arrows indicate morphological signatures of yeast isolates (*i.e.*, *Starmerella bombicola* L28; Supplementary Figure S8). Other (**d** and **e**) yeast-like and (**f**) fungal-like morphologies detected in the honey bee gut.

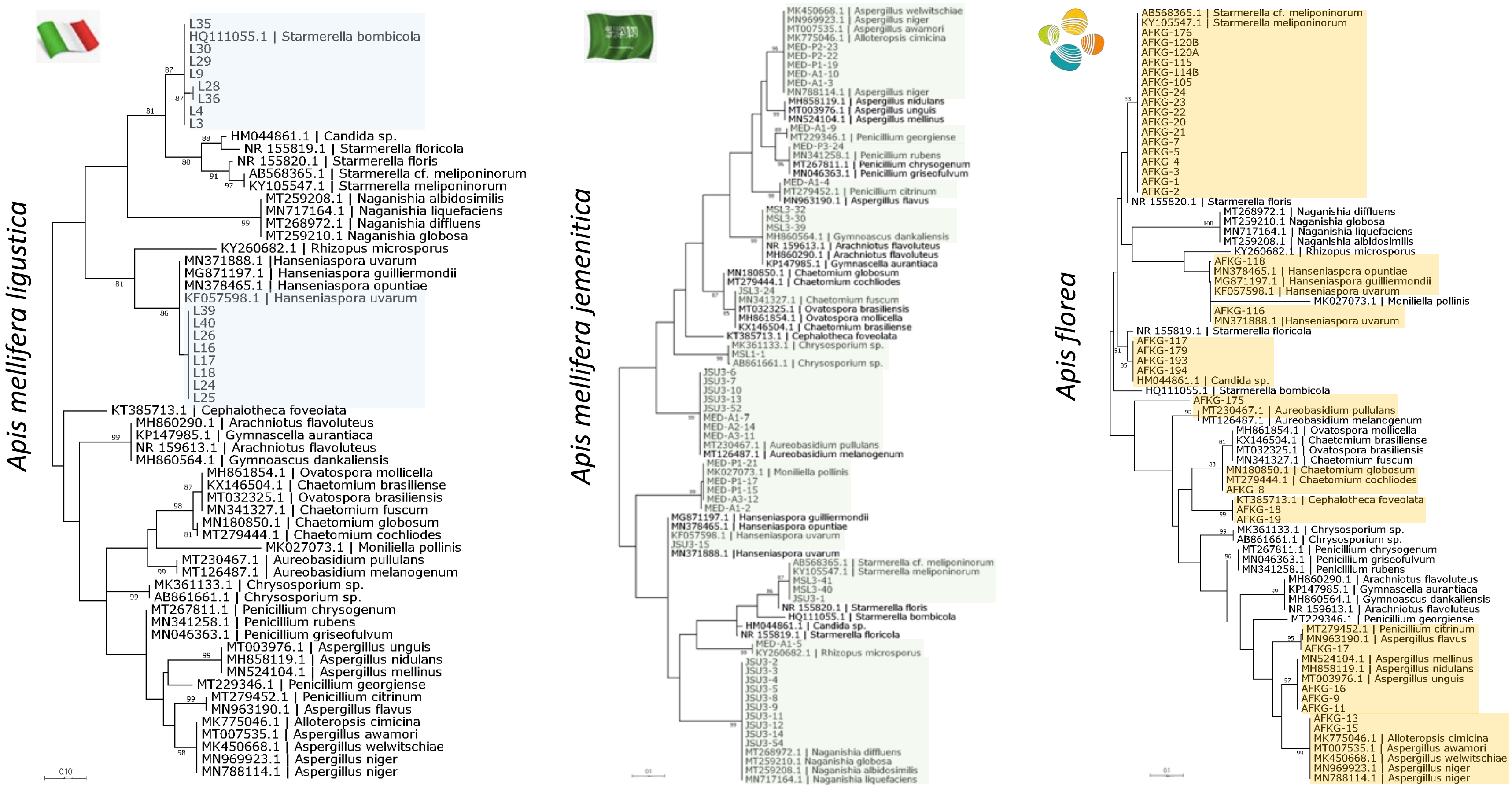

Supplementary Figure S8. The phylogenetic trees of the fungal isolates based on ITS sequences, and other fungal species relatives based on a neighbour-joining analysis of ITS sequences, from the gut of (**a**) the Italian honey bee *Apis mellifera ligustica*, (**b**) the indigenous Arabian honey bee *A. mellifera jemenitica*, and (**c**) the dwarf Arabian honey bee *A. florea*.

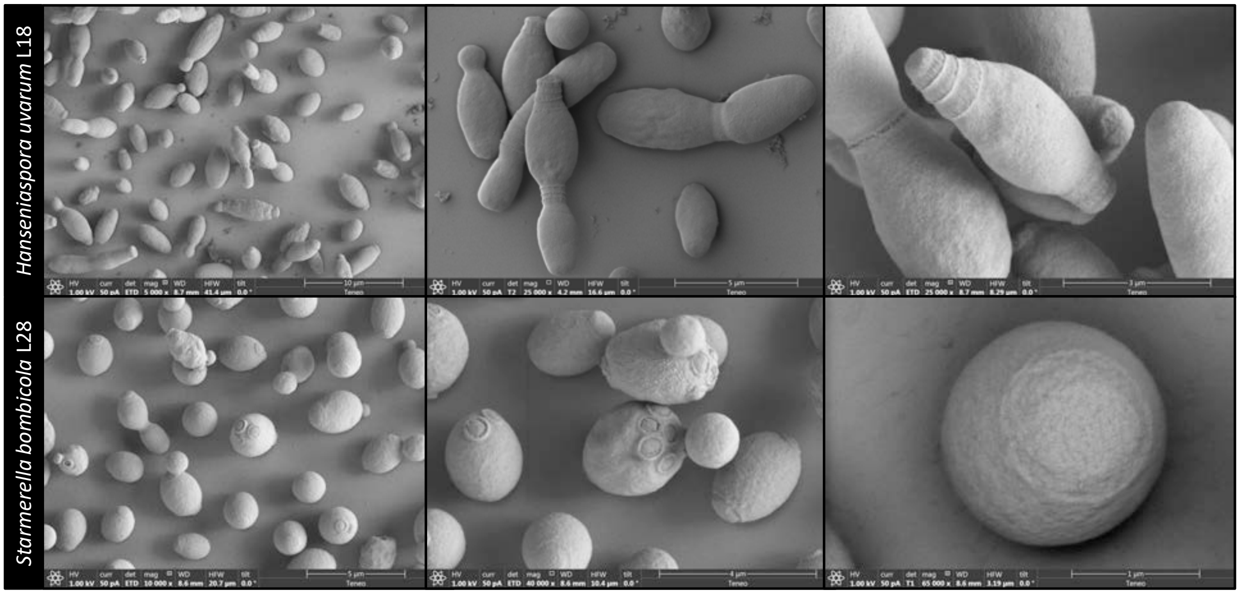

Supplementary Figure S9. Fungal isolates form the honeybee guts (Italian hive). Pure cultures of *Hanseniospora uvarum* isolate L18 (upper panels) and *Starmerella bombicola* isolate L28 (lower panels) were visualized at the SEM.

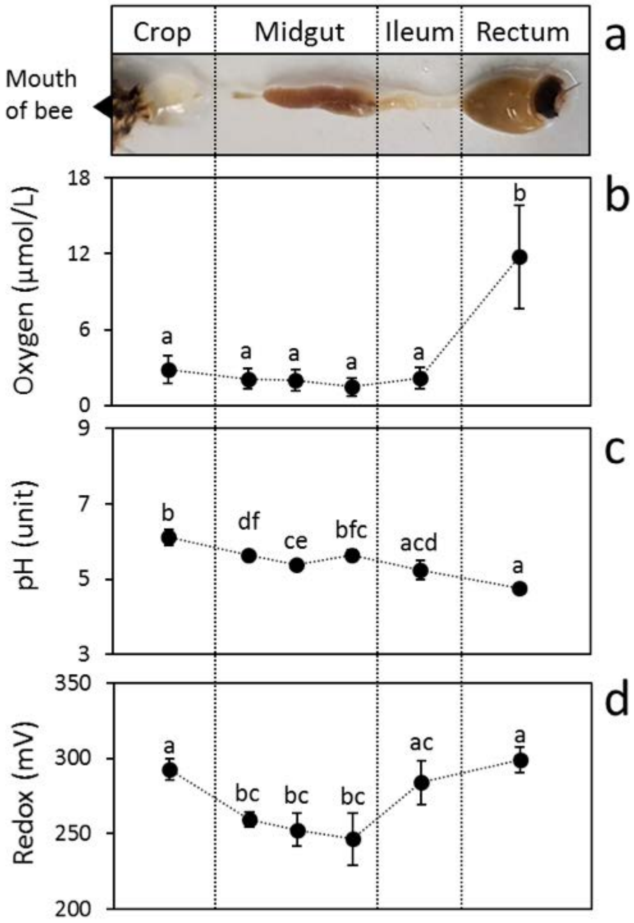

Supplementary Figure S10. Physicochemical conditions of oxygen (**b**), pH (**c**), redox potential (**d**) measured in all the gut tracts (**a**). Measures in the midgut were taken at three different points (anterior, middle, and posterior part). Values are expressed as mean ± error standard. Letters indicates statistical significance among the measures.

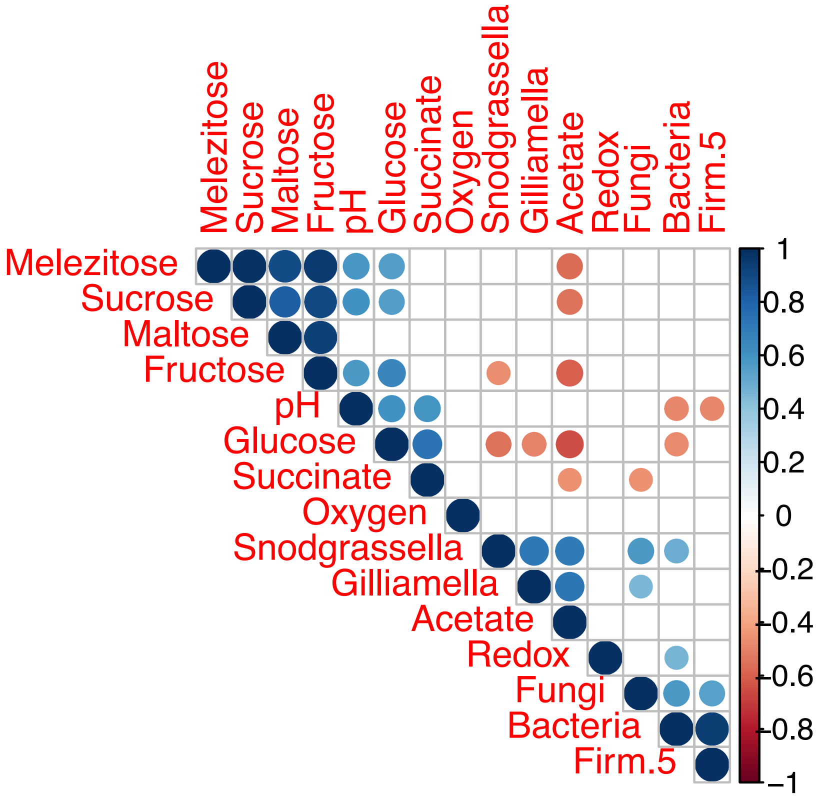

Supplementary Figure S11. Cross-correlogram showing the correlation among the physio-chemical variable and the bacterial and fungal abundance. The diagram shows only the significant Pearson correlation. Empty cells mean that the significance was less than 0.05. The intensity of the colour indicates the strength of the Pearson correlation among the variables.

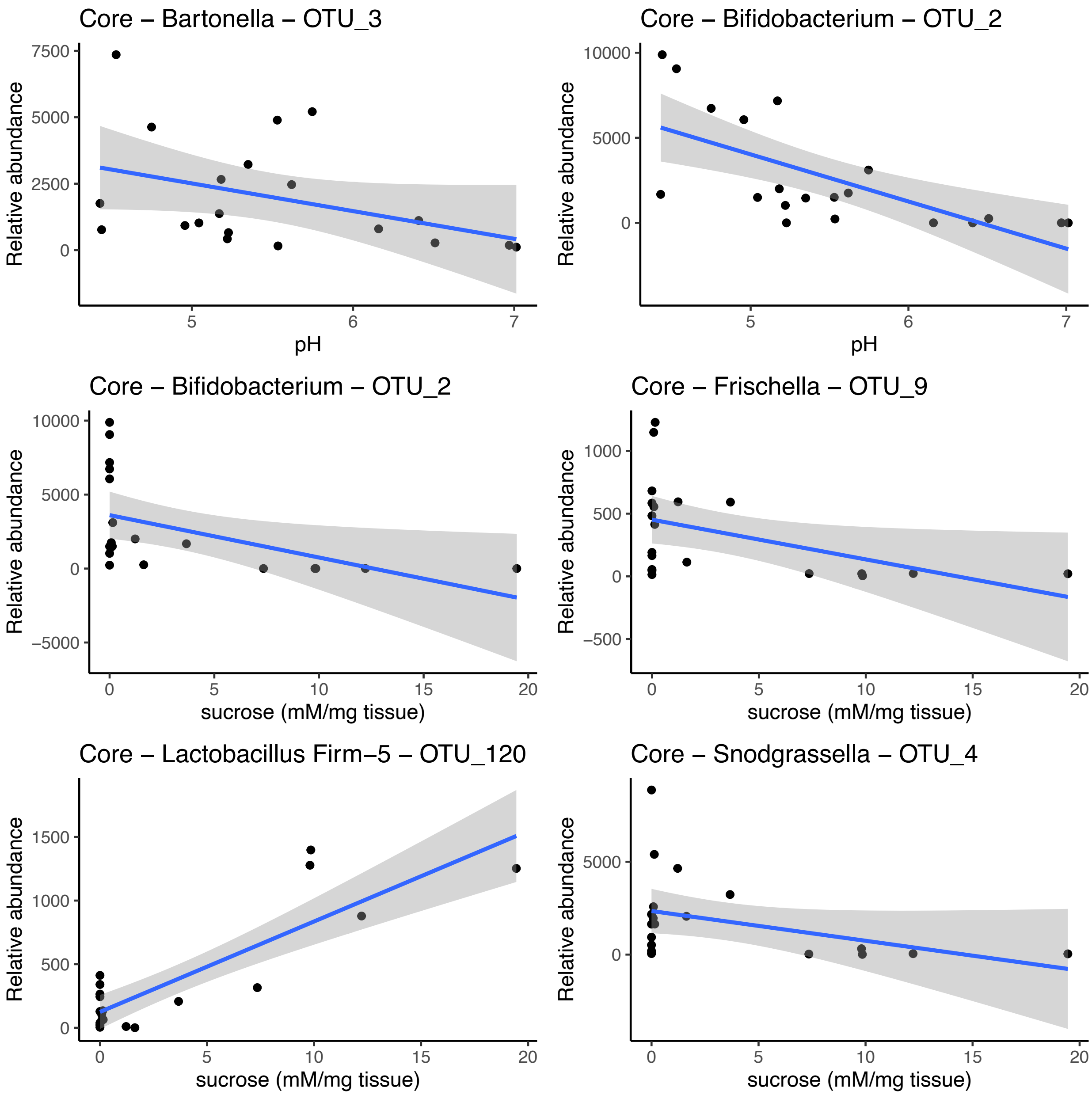

Supplementary Figure S12. Univariate generalised linear model describing the most significant changes in core bacterial OTUs abundance, along with the most important physico-chemical (pH) and metabolic (sucrose) explanatory variables that characterize the gut compartments.

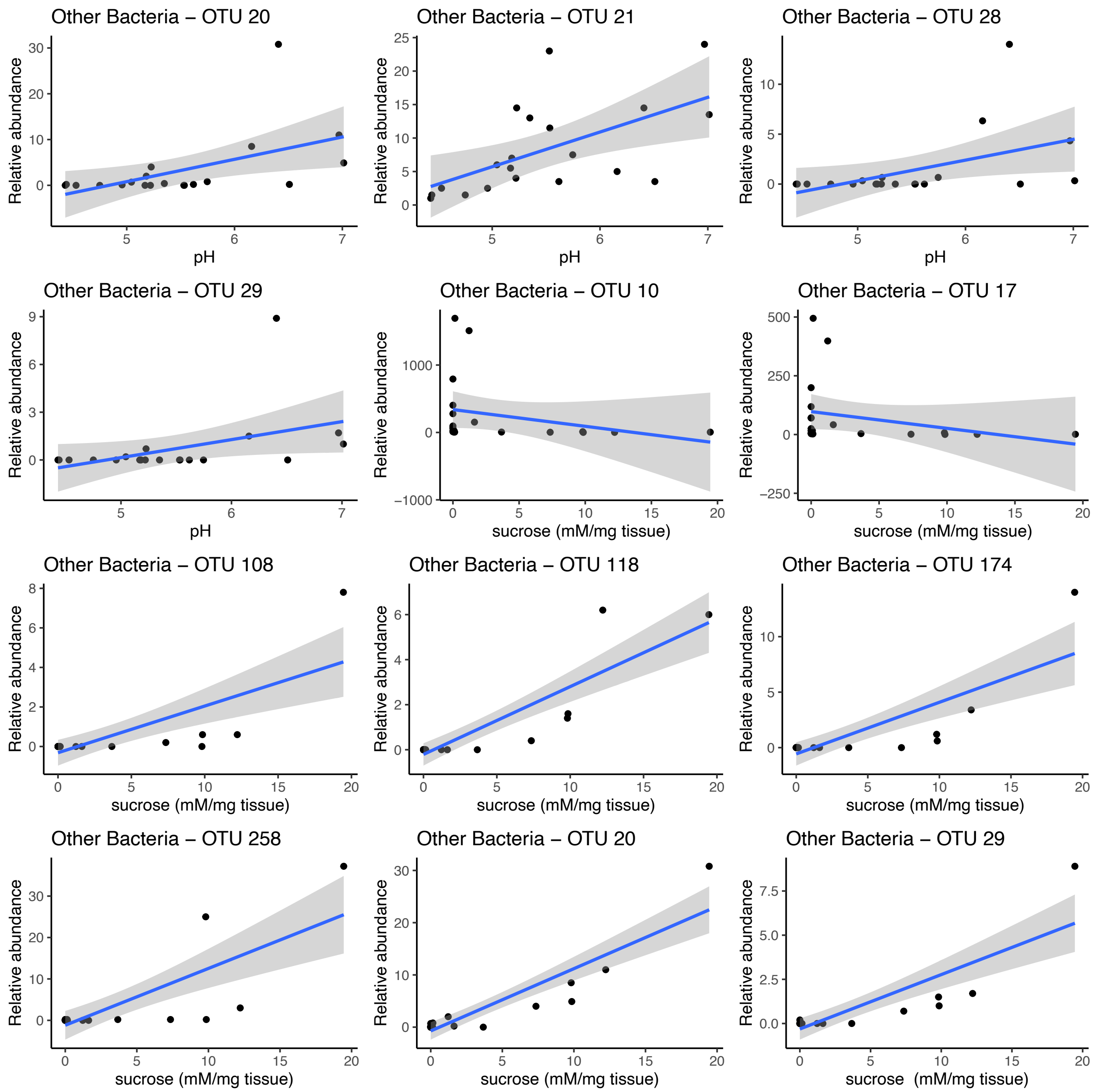

Supplementary Figure S13. Univariate generalised linear model describing the most significant changes in other-possibly bacterial OTUs abundance, along with the most important physico-chemical (pH) and metabolic (sucrose) explanatory variables that characterize the gut compartments.

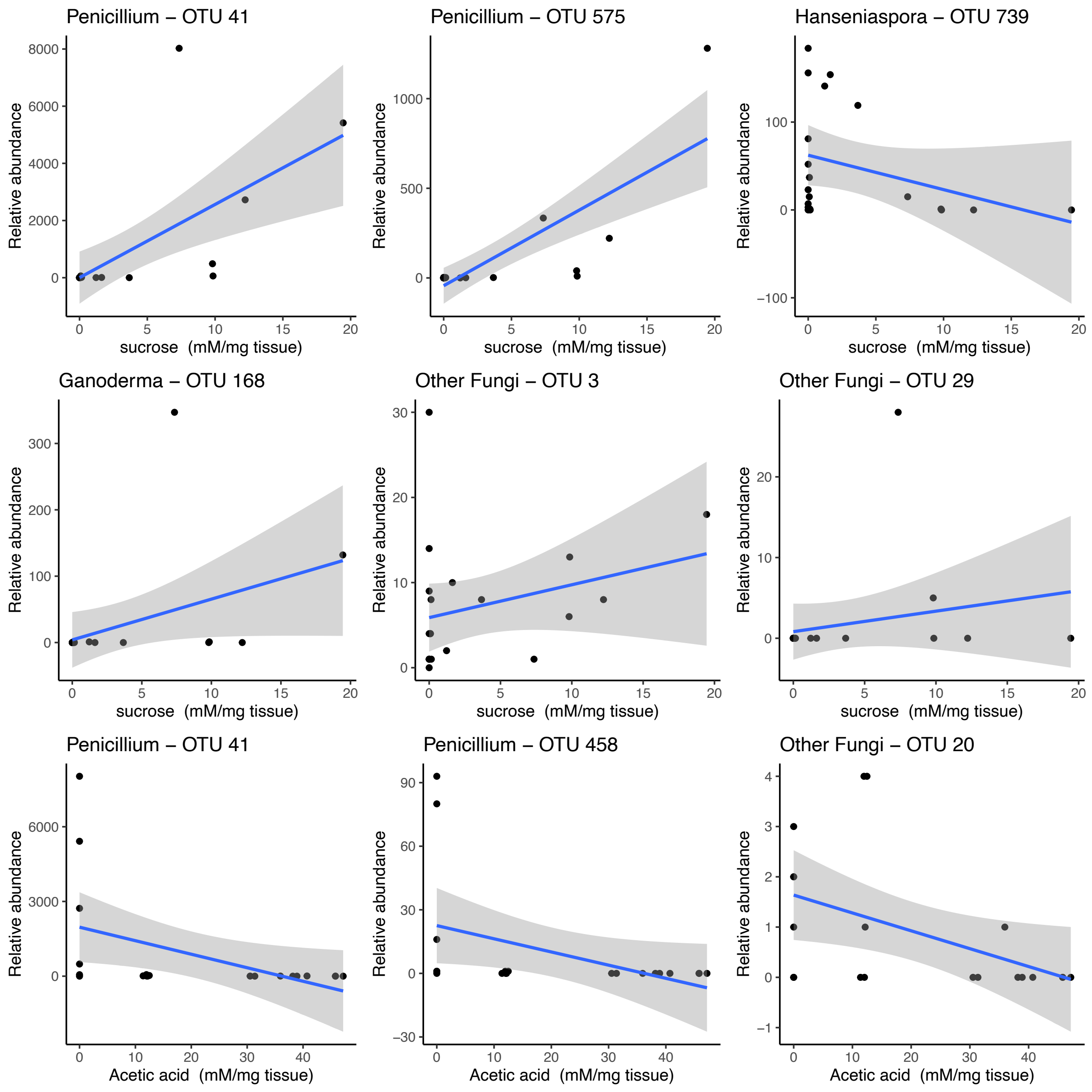

Supplementary Figure S14. Univariate generalised linear model describing the most significant changes in fungal OTUs abundance, along with the most important metabolic explanatory variables (sucrose and acetic acid) that characterize the gut compartments.

SUPPLEMENTARY DATA

Supplementary Data S1. Taxonomy and distribution of bacterial (amplified from DNA and cDNA) and fungal OTUs along the honey bee' gut compartments. Dataset from Italian (n = 5 pools per compartment) and Saudi Arabian (n = 5 pools per compartment) forager bees are reported. Normalization factors are also reported. Refer to the attached excel file named 'Callegari et al 2021_Supplementray Data S1.xlsx'.

Supplementary Data S2. Functional metabolic patterns interfered from the taxonomy of other-possibly environmental bacteria (cDNA) in the honey bee gut and fungal trophic categories obtained from fungal taxonomy. Refer to the attached excel file named 'Callegari et al 2021_Supplementray Data S2.xlsx'

Supplementary References

Babendreier, D., Joller, D., Romeis, J.J., Bigler, F., and Widmer, F. (2007) Bacterial community structures in honeybee intestines and their response to two insecticidal proteins. *FEMS Microbiol Ecol* **59**: 600–610.

Engel, P., Kwong, W.K., and Moran, N.A. (2013) Frischella perrara gen. nov., sp. nov., a gammaproteobacterium isolated from the gut of the honeybee, Apis mellifera. *IInternational J Syst Evol Microbiol* **63**: 3646–3651.

Jukes, T.H. and Cantor, C.R. (1969) Evolution of Protein Molecules. In *Mammalian Protein Metabolism*. Elsevier, pp. 21–132.

Kešnerová, L., Moritz, R., and Engel, P. (2016) Bartonella apis sp. nov., a honey bee gut symbiont of the class Alphaproteobacteria. *Int J Syst Evol Microbiol* **66**: 414–421.

Kumar, S., Stecher, G., Tamura, K., and Dudley, J. (2016) MEGA7: Molecular evolutionary genetics analysis version 7.0 for bigger datasets. *Mol Biol Evol* **33**: 1870–1874.

Kwong, W.K., Engel, P., Koch, H., and Moran, N.A. (2014) Genomics and host specialization of honey bee and bumble bee gut symbionts. *Proc Natl Acad Sci U S A* **111**: 11509–11514.

Kwong, W.K., Medina, L.A., Koch, H., Sing, K.-W., Soh, E.J.Y., Ascher, J.S., et al. (2017) Dynamic microbiome evolution in social bees. *Sci Adv* **3**: e1600513.

Leonhardt, S.D. and Kaltenpoth, M. (2014) Microbial communities of three sympatric Australian stingless bee species. *PLoS One* **9**: e105718.

Ludvigsen, J., Rangberg, A., Avershina, E., Sekelja, M., Kreibich, C., Amdam, G., and Rudi, K. (2015) Shifts in the Midgut/Pyloric Microbiota Composition within a Honey Bee Apiary throughout a Season. *Microbes Environ* **30**: 235–244.

Martinson, V.G., Danforth, B.N., Minkley, R.L., Rueppel, O., Tingek, S., and Moran, N.A. (2011) A simple and distinctive microbiota associated with honey bees and bumble bees. *Mol Ecol* **20**: 619–628.

Martinson, V.G., Moy, J., and Moran, N.A. (2012) Establishment of characteristic gut bacteria during development of the honeybee worker. *Appl Environ Microbiol* **78**: 2830–2840.

Siozios, S., Moran, J., Chege, M., Hurst, G.D.D., and Paredes, J.C. (2019) Complete reference genome assembly for Commensalibacter sp. strain AMU001, an acetic acid bacterium Isolated from the gut of honey bees. *Microbiol Resour Announc* **8**: 1–2.

Yun, J.-H., Lee, J.-Y., Hyun, D.-W., Jung, M.-J., and Bae, J.-W. (2017) Bombella apis sp. nov., an acetic acid bacterium isolated from the midgut of a honey bee. *Int J Syst Evol Microbiol* **67**: 2184–2188.

Zheng, H., Perreau, J., Powell, J.E., Han, B., Zhang, Z., Kwong, W.K., et al. (2019) Division of labor in honey bee gut microbiota for plant polysaccharide digestion. *Proc Natl Acad Sci* **116**: 25909–25916.

Zheng, H., Powell, J.E., Steele, M.I., Dietrich, C., and Moran, N.A. (2017) Honeybee gut microbiota promotes host weight gain via bacterial metabolism and hormonal signaling. *Proc Natl Acad Sci* **114**: 4775–4780.
