# Supplementary material for: Compartmentalization of bacterial and fungal microbiomes in the gut of adult honeybees
Source: NPJ Biofilms Microbiomes. 2021 May 7;7:42. doi: 10.1038/s41522-021-00212-9 (PMC8105395; doi:10.1038/s41522-021-00212-9)
Supplement: Supplementary file 4 — Reporting Summary [file 41522_2021_212_MOESM4_ESM.pdf]

nature research

Corresponding author(s): Daniele Daffonchio, Elena Crotti

Last updated by author(s): Mar 18, 2021

Reporting Summary

Nature Research wishes to improve the reproducibility of the work that we publish. This form provides structure for consistency and transparency in reporting. For further information on Nature Research policies, see Authors & Referees and the Editorial Policy Checklist.

Statistics

For all statistical analyses, confirm that the following items are present in the figure legend, table legend, main text, or Methods section.

n/a	Confirmed	
☐	☒	The exact sample size (*n*) for each experimental group/condition, given as a discrete number and unit of measurement
☐	☒	A statement on whether measurements were taken from distinct samples or whether the same sample was measured repeatedly
☐	☒	The statistical test(s) used AND whether they are one- or two-sided<br>*Only common tests should be described solely by name; describe more complex techniques in the Methods section.*
☐	☒	A description of all covariates tested
☐	☒	A description of any assumptions or corrections, such as tests of normality and adjustment for multiple comparisons
☐	☒	A full description of the statistical parameters including central tendency (e.g. means) or other basic estimates (e.g. regression coefficient) AND variation (e.g. standard deviation) or associated estimates of uncertainty (e.g. confidence intervals)
☒	☐	For null hypothesis testing, the test statistic (e.g. *F*, *t*, *r*) with confidence intervals, effect sizes, degrees of freedom and *P* value noted<br>*Give P values as exact values whenever suitable.*
☒	☐	For Bayesian analysis, information on the choice of priors and Markov chain Monte Carlo settings
☒	☐	For hierarchical and complex designs, identification of the appropriate level for tests and full reporting of outcomes
☒	☐	Estimates of effect sizes (e.g. Cohen's *d*, Pearson's *r*), indicating how they were calculated

Our web collection on statistics for biologists contains articles on many of the points above.

Software and code

Policy information about availability of computer code

Data collection	Unisense Sensor Trace Suite
Data analysis	UPARSE v8, QIIME 1.9, Gold, UClast, SILVA 123, UNITE, R v 3.5, Primer 6, Prism 8

For manuscripts utilizing custom algorithms or software that are central to the research but not yet described in published literature, software must be made available to editors/reviewers. We strongly encourage code deposition in a community repository (e.g. GitHub). See the Nature Research guidelines for submitting code & software for further information.

Data

Policy information about availability of data

All manuscripts must include a data availability statement. This statement should provide the following information, where applicable:

- Accession codes, unique identifiers, or web links for publicly available datasets
- A list of figures that have associated raw data
- A description of any restrictions on data availability

Raw data described in this manuscript are submitted in Sequence Read Archive (SRA) of NCBI under the BioProject accession number PRJNA422176, PRJNA632549, and PRJNA422177. Sequences of fungal isolates are submitted in the European Nucleotide Archive (ENA) under the accession numbers LR746502-LR746517 and LR798000-LR798072.

Field-specific reporting

Please select the one below that is the best fit for your research. If you are not sure, read the appropriate sections before making your selection.

☐ Life sciences ☐ Behavioural & social sciences ☒ Ecological, evolutionary & environmental sciences

For a reference copy of the document with all sections, see nature.com/documents/nr-reporting-summary-flat.pdf

Ecological, evolutionary & environmental sciences study design

All studies must disclose on these points even when the disclosure is negative.

Study description	The goal of the study was to deepen the knowledge on the diversity of variable–less abundant–environmental bacterial phylotypes and neglected fungal members that accompanied the recurring bacterial phylotypes of eusocial corbiculate bee gut core microbiome. We used hives from Italy and Saudi Arabia to collect honey bee workers in different years and characterize the total and active bacterial community, and total fungal community associated with the gut compartments. Presence of microbial components (bacteria and fungi) in the gut was confirmed using electron microscopy. Viability of fungal cells was also proved performing in vitro isolation.
Research sample	Gut compartments of honey bee workers were collected from Italy (1 location) and Saudi Arabia (4 locations)
Sampling strategy	Honey bee worker were randomly sampled during flights outside the hives.
Data collection	All the honey bees collected in the field were bring to the laboratory and visualized at the stereo-microscope in order to identify the species and subspecies before to proceed with dissection and extraction of intestinal traits.<br>Data collection on gut (i.e., physic-chemical parameters) was performed in the laboratories at the University of Milan and KAUST by MC, EC, EG with the support of the honeybee keepers (listed in the acknowledgment of the manuscript).
Timing and spatial scale	The sampling of the honey bees was performed in Italy in September 2016 and October 2018, and in Saudi Arabia in January and February 2020.
Data exclusions	Fungal sequencing dataset from midgut pool 1 were excluded form the analysis due to low number of reads.
Reproducibility	All the finding can be replicated as all the techniques used may be reproduced. Micro-profiling of physico-chemical conditions of gut compartments were obtained using UniSense micro-sensors following protocols described in the material and methods. On the other hand microbial biomass and diversity was determined using qPCR and MiSeq sequencing that can be easily replicated following the procedure in the Methods Section.
Randomization	Environmental honey bees used in the experiment to obtain the intestinal tracts were all workers and were not allocated into experimental groups. They were only grouped by gut compartments during the statistical analyses to evaluate the compartmentalization effect of gut.
Blinding	Blinding did not apply to our study because it did not involve human participants or any parameters that were manually recorded and could be biased by the human researchers.

Did the study involve field work? ☒ Yes ☐ No

Field work, collection and transport

Field conditions	Italy (Garugliasco, Piedmont) and Saudi Arabia (Jeddah, Bryman, Al Musayjid and KAUST) fields hosting honey bee hives.
Location	IT - Grugliasco, Piedmont: Latitude 45.066874, Longitude 7.590442<br>KSA - Jeddah, Makkah: Latitude 21.484389, Longitude 39.194639<br>KSA - Bryman, Makkah: Latitude 21.647138, Longitude 39.248959<br>KSA - Al Musayjid, Madinah: Latitude 24.087309, Longitude 39.095556<br>KSA - KAUST (Thuwal), Makkah: Latitude 22.326796, Longitude 39.109266
Access and import/export	All necessary permits were in hand when the research was conducted in Italy and Saudi Arabia, and the research was done in compliance of national (Italy and Saudi Arabia) and International guidelines.
Disturbance	Collection of honey bees workers from the hives was performed minimizing the disturbance to the colonies.

Reporting for specific materials, systems and methods

We require information from authors about some types of materials, experimental systems and methods used in many studies. Here, indicate whether each material, system or method listed is relevant to your study. If you are not sure if a list item applies to your research, read the appropriate section before selecting a response.

Materials & experimental systems

n/a	Involved in the study	
☒	☐	Antibodies
☒	☐	Eukaryotic cell lines
☒	☐	Palaeontology
☐	☒	Animals and other organisms
☒	☐	Human research participants
☒	☐	Clinical data

Methods

n/a	Involved in the study	
☒	☐	ChIP-seq
☒	☐	Flow cytometry
☒	☐	MRI-based neuroimaging

Animals and other organisms

Policy information about studies involving animals; ARRIVE guidelines recommended for reporting animal research

Laboratory animals	The study did non involve laboratory animals.
Wild animals	Adult worker bees were collected from the front of the hive using a clear, widemouthed, well ventilated transparent sterile container with numerous holes. The collected honey bees were transported in the laboratory, checked at the stereo-microscope and further chilled in a sterile petri dish at 4°C for 10 minutes before dissection and collection of intestinal trait.
Field-collected samples	All the specimens collected were immediately transported in laboratory and processed. Insect were not stored or incubated in laboratory condition.
Ethics oversight	No ethics approval is foreseen for bees since they are not included in the higher invertebrate category. Animals used in our experiments were maintained and treated in compliance with the guidelines specified by the Italian Ministry of Forestry and Agriculture and Saudi Arabia regulations.

Note that full information on the approval of the study protocol must also be provided in the manuscript.
